# Very-Low-Density Lipoprotein–Associated Apolipoproteins Predict Cardiovascular Events and Are Lowered by Inhibition of APOC-III

**DOI:** 10.1016/j.jacc.2016.11.065

**Published:** 2017-02-21

**Authors:** Raimund Pechlaner, Sotirios Tsimikas, Xiaoke Yin, Peter Willeit, Ferheen Baig, Peter Santer, Friedrich Oberhollenzer, Georg Egger, Joseph L. Witztum, Veronica J. Alexander, Johann Willeit, Stefan Kiechl, Manuel Mayr

**Affiliations:** a Department of Neurology, Medical University Innsbruck, Innsbruck, Austria; b University of California San Diego, La Jolla, California; c Ionis Pharmaceuticals, Carlsbad, California; d King’s British Heart Foundation Centre, King’s College London, London, United Kingdom; e Department of Public Health and Primary Care, University of Cambridge, Cambridge, United Kingdom; f Department of Laboratory Medicine, Hospital of Bruneck, Bruneck, Italy; g Department of Internal Medicine, Hospital of Bruneck, Bruneck, Italy

**Keywords:** lipidomics, mass spectrometry, proteomics, triglycerides, apo, apolipoprotein, CE, cholesterol ester, CVD, cardiovascular disease, DAG, diacylglycerol, FCS, familial chylomicronemia syndrome, HDL, high-density lipoprotein, HDL-C, high-density lipoprotein cholesterol, LDL, low-density lipoprotein, LDL-C, low-density lipoprotein cholesterol, MRM, multiple reaction monitoring, MS, mass spectrometry, PC, phosphatidylcholine, TAG, triacylglycerol, TG, triglyceride, TRL, triglyceride-rich lipoprotein, VLDL, very-low-density lipoprotein

## Abstract

**Background:**

Routine apolipoprotein (apo) measurements for cardiovascular disease (CVD) are restricted to apoA-I and apoB. Here, the authors measured an unprecedented range of apolipoproteins in a prospective, population-based study and relate their plasma levels to risk of CVD.

**Objectives:**

This study sought to measure apolipoproteins directly by mass spectrometry and compare their associations with incident CVD and to obtain a system-level understanding of the correlations of apolipoproteins with the plasma lipidome and proteome.

**Methods:**

Associations of 13 apolipoproteins, 135 lipid species, and 211 other plasma proteins with incident CVD (91 events), defined as stroke, myocardial infarction, or sudden cardiac death, were assessed prospectively over a 10-year period in the Bruneck Study (N = 688) using multiple-reaction monitoring mass spectrometry. Changes in apolipoprotein and lipid levels following treatment with volanesorsen, a second-generation antisense drug targeting apoC-III, were determined in 2 human intervention trials, one of which was randomized.

**Results:**

The apolipoproteins most significantly associated with incident CVD were apoC-II (hazard ratio per 1 SD [HR/SD]: 1.40; 95% confidence interval [CI]: 1.17 to 1.67), apoC-III (HR/SD: 1.38; 95% CI: 1.17 to 1.63), and apoE (HR/SD: 1.31; 95% CI: 1.13 to 1.52). Associations were independent of high-density lipoprotein (HDL) and non-HDL cholesterol, and extended to stroke and myocardial infarction. Lipidomic and proteomic profiles implicated these 3 very-low-density lipoprotein (VLDL)-associated apolipoproteins in de novo lipogenesis, glucose metabolism, complement activation, blood coagulation, and inflammation. Notably, apoC-II/apoC-III/apoE correlated with a pattern of lipid species previously linked to CVD risk. ApoC-III inhibition by volanesorsen reduced plasma levels of apoC-II, apoC-III, triacylglycerols, and diacylglycerols, and increased apoA-I, apoA-II, and apoM (all p < 0.05 vs. placebo) without affecting apoB-100 (p = 0.73).

**Conclusions:**

The strong associations of VLDL-associated apolipoproteins with incident CVD in the general community support the concept of targeting triacylglycerol-rich lipoproteins to reduce risk of CVD.

Alterations in lipid metabolism underlie atherosclerotic cardiovascular disease (CVD) (1). The low-density lipoprotein (LDL)–cholesterol axis is an established target in CVD prevention. In contrast to genetic disorders that lead to higher or lower LDL cholesterol (LDL-C), genetic polymorphisms that affect high-density lipoprotein (HDL) cholesterol (HDL-C) have not consistently translated into altered cardiovascular risk. Thus far, explanations for CVD risk have primarily focused on quantities of only a few lipids within established lipoprotein classes, such as LDL-C and very low-density lipoprotein (VLDL) triglycerides (TGs), and, for the most part, ignored other lipid species (1). Yet individual molecular lipid species within the same lipid class display different associations with CVD (1). Although lipoproteins are defined by their flotation properties during ultracentrifugation, their functions and metabolism are principally governed by their apolipoprotein content. However, no comprehensive analysis of plasma apolipoproteins and lipids has been performed in the same cohort to assess their comparative association with future CVD in the general community.

Here, we capitalized on recent advances in mass spectrometry (MS) by applying multiple-reaction monitoring MS (MRM-MS) to the prospective, population-based Bruneck Study. We measured 13 plasma apolipoproteins by use of spiked-in, stable isotope–labeled standards, integrated the apolipoprotein panel with clinical, proteomic, and lipidomic measurements, and analyzed their predictive value for CVD. Unexpectedly, after multivariate analysis, VLDL-associated apolipoproteins and predominant lipids emerged as the strongest determinants of CVD risk. We also show that these VLDL-associated apolipoproteins and their corresponding lipid species can be reduced by targeting apolipoprotein C-III (apoC-III), a central regulator of plasma triglyceride-rich lipoprotein (TRL) metabolism 2, 3.

## Methods

An expanded Methods section is available in the Online Appendix. Associations of 13 apolipoproteins, 135 lipid species, and 211 other plasma proteins with incident CVD were assessed prospectively over a 10-year period in the Bruneck Study. Changes in apolipoprotein and lipid levels following treatment with volanesorsen, a second-generation antisense drug targeting apoC-III, were determined in 2 human intervention trials (IONIS1 and IONIS2), one of which was randomized.

### Bruneck study

The Bruneck Study is a prospective, population-based survey of the epidemiology and pathogenesis of atherosclerosis and CVD (1). An age- and sex-stratified random sample of all inhabitants of Bruneck, Italy, all of Caucasian descent, was enrolled in 1990. In 2000, 702 subjects were still alive, and participated in the second quinquennial follow-up. Measurements taken in 2000 served as the baseline for the present study. Detailed information on fatal and nonfatal CVD was carefully collected for these subjects until 2010, with follow-up 100% complete for clinical outcomes. Clinical outcomes were adjudicated by 1 senior researcher blinded to baseline data.

The study protocol was approved by the ethics committees of Bolzano and Verona, and conformed to the Declaration of Helsinki, and all study subjects gave written informed consent. The composite CVD endpoint included incident fatal and nonfatal myocardial infarction, ischemic stroke, and sudden cardiac death. The presence of myocardial infarction was assessed by World Health Organization criteria (4), and ischemic stroke was classified according to the criteria of the National Survey of Stroke (5).

### MRM-MS in plasma

Citrate plasma samples were stored at −80°C until analysis. Upon thawing, peptide standards were spiked into the samples (PlasmaDive kits, Biognosys AG, Schlieren, Switzerland). The peptide standard for apoB-100 did not overlap with the proximal portion of apoB that would include both apoB-48 and apoB-100. An appropriate apo(a) standard was not available, and thus apo(a) levels were not measured. Plasma samples were processed according to the manufacturer’s instructions. Briefly, 10 μl of plasma samples were denatured, reduced, and alkylated. Proteins (20 μg) were spiked with authentic heavy peptide standards. An in-solution digestion was performed overnight. After solid-phase extraction with C18 spin columns (96-well format, Harvard apparatus, Holliston, Massachusetts), the eluted peptides were dried using a SpeedVac (ThermoFisher Scientific, Woburn, Massachusetts) and resuspended in 40 μl of liquid chromatography solution. The samples were analyzed on an Agilent 1290 liquid chromatography system (Agilent Technologies, Santa Clara, California) interfaced to an Agilent 6495 Triple Quadrupole MS (Agilent Technologies). Samples (10 μl) were directly injected onto a 25-cm column (AdvanceBio Peptide Map 2.1 × 250 mm, Agilent Technologies) and separated over a 23-min gradient at 350 μl/min. Data were analyzed using Skyline software version 3.1 (MacCoss Lab, University of Washington, Seattle, Washington) and protein concentrations were calculated using the heavy/light (H/L) ratio.

### Statistical analysis

Baseline characteristics are presented as count (percentage), mean ± SD, or median (interquartile range). Associations with incident endpoints were examined using Cox regression. The proportional hazards assumption was tested using the correlation of Schoenfeld residuals with survival time, and was not refuted. Cox models were progressively adjusted for age, sex, and statin therapy (model 1), plus diabetes mellitus, current smoking, and systolic blood pressure (model 2), plus HDL-C and non–HDL-C (model 3).

Cross-sectional analyses on clinical variables and on lipidomic data employed linear regression, adjusted for age, sex, and statin therapy. Correlation analyses used Pearson correlation partial to age, sex, and statin therapy, and log-transformed proteins if their skewness exceeded 2. Lipid variables were clustered using agglomerative hierarchical clustering on the basis of complete linkage, defining the distance between 2 variables as 1 minus their correlation. Cross-sectional analyses of high-dimensional protein data deemed associations significant according to a false discovery rate q value below 0.05. Other results were not adjusted for multiplicity (6).

To estimate effects of apoC-III synthesis inhibition, for each subject, measurements at day 1 (baseline) and means of measurements at days 57 and 92 (under treatment) were log-transformed, and the change from baseline was calculated as their difference. The mean change from baseline in each group (IONIS1 treated, IONIS2 treated, IONIS2 placebo) was tested by 1-sample Student *t* tests against a mean of 0. For presentation of effect sizes, the mean change from baseline was transformed from the log scale to a percent scale. Differential changes from baseline in the IONIS2-treated and placebo groups were tested using Mann-Whitney-Wilcoxon tests. The incremental predictive value provided by apolipoprotein measurements was investigated as described in the Online Appendix. Analyses were conducted using R 3.2.0 (R Project for Statistical Computing, Vienna, Austria). The p values are 2-sided, and an alpha level of 0.05 is used.

## Results

### Associations of baseline apolipoproteins and lipids with CVD

**Figure 1:**
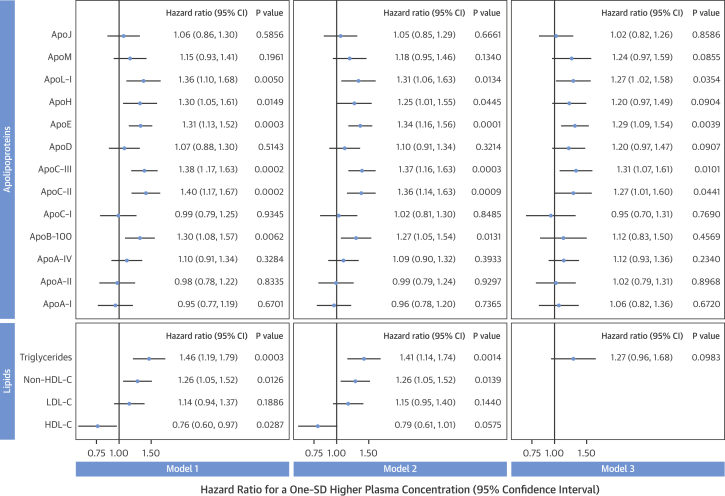
Associations of Apolipoproteins and Lipid Measures With Incident CVD Plasma levels of 13 apolipoproteins and of 4 conventional lipid measures were determined in 688 participants of the Bruneck Study. During 10 years of follow-up, 91 cardiovascular events occurred, comprising stroke, myocardial infarction, and sudden cardiac death. **Model 1**: Adjustment for age, sex, and statin therapy. **Model 2**: As in model 1, with additional adjustment for diabetes, systolic blood pressure, and smoking. **Model 3**: As in model 2, with additional adjustment for HDL-C and non–HDL-C. Quantitatively, for each variable, 1 SD corresponds to: ApoA-I, 607 mg/l; ApoA-II, 6.44 mg/l; ApoA-IV, 15.0 mg/l; ApoB-100, 363 mg/l; ApoC-I, 6.46 mg/l; ApoC-II, 6.30 mg/l; ApoC-III, 25.6 mg/l; ApoD, 7.98 mg/l; ApoE, 9.23 mg/l; ApoH, 38.2 mg/l; ApoL-I, 3.93 mg/l; ApoM, 2.42 mg/l; ApoJ, 23.1 mg/l; HDL-C, 15.2 mg/dl; LDL-C, 36.5 mg/dl; non-HDL-C, 41.4 mg/dl; triglycerides, 77.6 mg/dl. apo = apolipoprotein; CI = confidence interval; CVD = cardiovascular disease; HDL-C = high-density lipoprotein cholesterol; LDL-C = low-density lipoprotein cholesterol.

Associations of apolipoproteins with incident CVD (2000 to 2010) were investigated in the population-based Bruneck Study (N = 688). Baseline clinical characteristics are summarized in Online Table 1. Subjects were on average 66 years old, 52% were female, 6.4% reported prior CVD, and 9% were prescribed statins. Among 13 apolipoproteins quantified by MRM-MS, the most significant associations with incident CVD were detected for apoC-II, apoC-III, and apoE (p < 0.001 each, under adjustment for age, sex, and statin therapy) (Figure 1, model 1), followed by apoL-I, apoB-100, and apoH (p ≤ 0.01 each). Additional adjustment for diabetes, systolic blood pressure, and current smoking did not appreciably alter these associations (Figure 1, model 2), but further adjustment for HDL-C and non–HDL-C rendered apoB-100 and apoH nonsignificant, and weakened the associations obtained for apoC-III, apoC-II, and apoE (Figure 1, model 3). The association of TGs with CVD (p < 0.001) also lost significance after adjustment for HDL-C and non–HDL-C (Figure 1). Similar results were obtained for the individual endpoints of stroke and myocardial infarction (Online Figures 1A and 1B, respectively). ApoL-I displayed a strong association specifically with stroke (Figure 1, Online Figures 1A and 1B). Upon exclusion of subjects with prior CVD (Online Figure 2) or of subjects prescribed statins (Online Figure 3), results did not change appreciably.

When investigating whether apoC-III, apoC-II, and apoE could improve on traditional risk factors in 10-year cardiovascular risk prediction (Online Table 2), no significant change in the c-index was found; however, a significantly positive net reclassification index indicated that 12.3% of subjects could be more appropriately classified into the clinically relevant risk categories of 0.0% to 5.0%, 5.0% to 7.5%, or more than 7.5% when including apolipoproteins.

### Interrelationships between apolipoproteins

**Figure 2:**
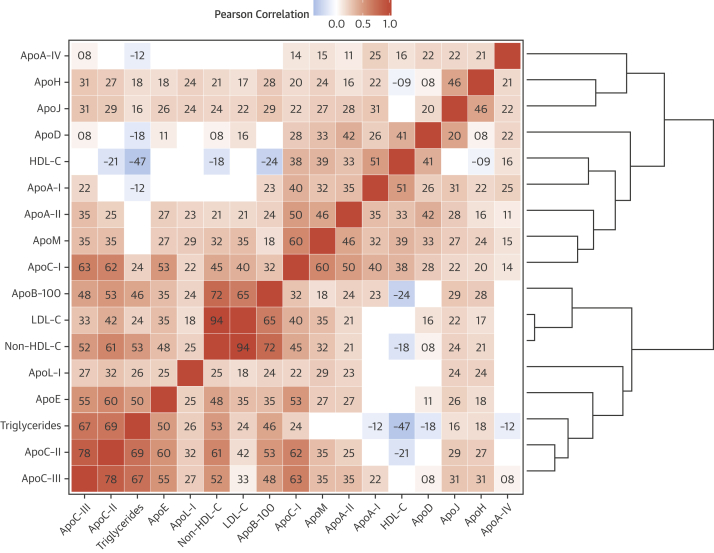
Correlations Among Apolipoproteins and Lipids Results are adjusted for age, sex, and statin therapy. **Tile color** codes for direction and magnitude of correlation, whereas tile text gives its sign and the first 2 decimal digits. Variables are arranged by similarity, as shown in the **right-hand dendrogram**. Only significant correlations are shown. Clustering gave rise to several groups of highly intercorrelated variables. High-level clusters were characterized by more extensive correlations with apoA-I **(top right region)** or with apoB-100 **(bottom left region)**, with the latter containing subclusters likely representing VLDL (including apoC-III, apoC-II, apoE, TG, non–HDL-C, and HDL-C) and LDL (including apoB-100 and LDL-C). VLDL = very-low-density lipoprotein; other abbreviations as in Figure 1.

**Figure 3:**
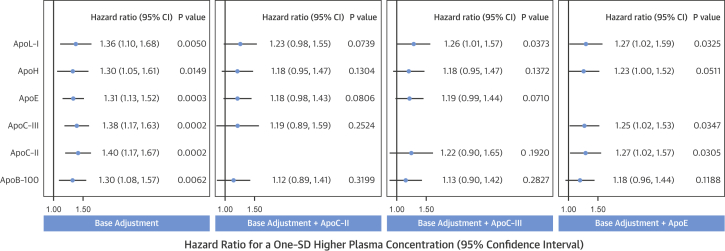
Associations of Apolipoproteins With Incident CVD Upon Additional Adjustment for ApoC-II, ApoC-III, or Apo-E Base adjustment consisted of adjustment for age, sex, and statin therapy and is shown for the significant apolipoproteins only in the first column (as in Figure 1). Additional adjustment for apoC-II, apoC-III, or apoE is shown in the other 3 columns, respectively. Note that apoB-100 loses its association with incident CVD upon adjustment for any of the 3 VLDL-associated apolipoproteins. Abbreviations as in Figures 1 and 2.

Correlations among apolipoproteins and standard lipid measures are shown in Figure 2. ApoC-II, apoC-III, apoE, and TGs formed one set of highly intercorrelated variables along with non–HDL-C (which represents VLDL cholesterol [VLDL-C] and LDL-C) and apoB-100 (primarily representative of LDL) (Figure 2, lower left quadrant). Another cluster comprised variables generally more correlated with apoA-I (Figure 2, upper right quadrant). ApoH and apoJ showed moderate correlations with most other variables. ApoC-I, which is known to primarily associate with VLDL, correlated most strongly with the apoB-100 cluster, and more weakly with apoA-I and HDL-C. Given the interrelationships among apolipoproteins, the hazard ratios for apolipoproteins significantly associated with CVD were adjusted for apoC-II, apoC-III, and apoE (Figure 3). Adjustment for apoE weakened the associations of apolipoproteins with CVD, in particular for apoB-100, whereas adjustment for apoC-II or apoC-III rendered all associations nonsignificant.

After adjustment for age, sex, and statin therapy, apoC-II, apoC-III, and apoE were related to several environmental and anthropological parameters known to be correlated with TG values, such as body mass index, waist-hip ratio, blood pressure, and alcohol consumption, and metabolic parameters, such as liver function tests, but surprisingly, only weakly to fasting plasma glucose and HbA_1c_. As expected, they related strongly to TGs, total cholesterol, LDL-C and non–HDL-C, with apoC-II and apoC-III showing stronger associations than apoE (Online Figure 4). Among 211 plasma proteins, apoC-II, apoC-III, and apoE showed correlations for proteins involved in lipid metabolism, blood coagulation, the complement system, or inflammation and immunity (Online Figures 5A to 5C, respectively), many of which were correlated to the liver-specific microRNA, miR-122, indicating a common hepatic origin.

### Interrelationships between apolipoproteins and plasma lipids

**Figure 4:**
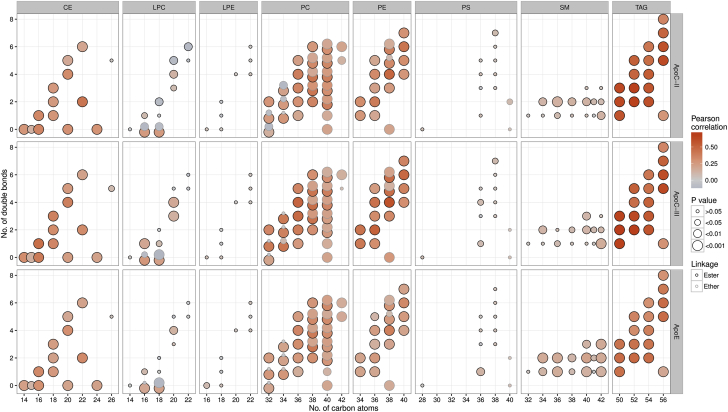
Associations of 135 Plasma Lipid Species With ApoC-II, ApoC-III, or ApoE Individual lipid species are depicted by **filled circles** and arranged by lipid class in 8 panels according to the number of total carbon atoms (x-axes) and number of double bonds (y-axes). **Circle fill color** represents the correlation of each lipid species with plasma concentrations of apoC-II, apoC-III, and apoE. Lipids with the same number of carbon atoms and double bonds are pulled apart vertically to increase their visibility. The distinguishing feature in this case is the presence of an alkyl ether linkage, signified in the formula as, for example, PC(O-38:3). Lipids possessing such a linkage are pulled upward, and their alkyl-ether-free counterparts are pulled downward. CE = cholesteryl ester; CI = confidence interval; LPC = lysophosphatidylcholine; LPE = lysophosphatidylethanolamine; PC = phosphatidylcholine; PE = phosphatidylethanolamine; PS = phosphatidylserine; SM = sphingomyelin; TAG = triacylglycerol.

The correlations of 135 molecular lipid species with apoC-II, apoC-III, and apoE are presented in Figure 4. Lipid species are represented by circles, with their position in the 2-dimensional lipid class graphs determined by their total acyl chain carbon numbers (x-axis) and double-bond content (y-axis). Circle color represents strength and direction (positive or negative) of correlations, and circle size indicates their level of statistical significance. ApoC-II, apoC-III, and apoE showed strong direct associations with cholesterol esters (CEs), phosphatidylcholines (PCs), phosphatidylethanolamines (PEs), and, particularly, triacylglycerols (TAGs).

The pattern of lipid species related to these 3 apolipoproteins resembled the adverse lipid signature of CVD that we recently described in the Bruneck Study (1), where associations with CVD risk were most pronounced for TAGs and CEs of lower carbon number and double-bond content (i.e., saturated and monounsaturated fatty acids), and the risk profile was complemented by PEs/PCs, sphingomyelins (both positive), and lysophosphatidylcholines (negative) (Online Figure 6, middle row). Of note, associations with CVD of most lipid species were substantially attenuated upon adjustment for apoC-II and apoC-III (Online Figures 6A and 6B, bottom row) and, to a lesser extent, upon adjustment for apoE (Online Figure 6C). This is consistent with the notion that apoC-II and apoC-III were more strongly correlated with total cholesterol, non–HDL-C, and TGs than apoE (Online Figure 4).

### Effect of lowering apoC-III levels on apolipoproteins and lipids in plasma

The data presented thus far support the hypothesis that VLDL and their associated lipids and apolipoproteins are atherogenic. It would be of considerable interest, therefore, to examine the consequences of lowering VLDL levels on plasma apolipoproteins and relevant lipids. Most hypolipidemic agents that have been used to lower VLDL levels directly target multiple lipoprotein levels and metabolism. However, antisense therapy is emerging as a novel lipid-lowering strategy, because it can specifically target the synthesis of a single apolipoprotein, and thus enable an examination of the consequences on the entire apolipoprotein and lipid profile. As noted earlier in the text, apoC-III has emerged as a central regulator of TRL metabolism.

**Figure 5:**
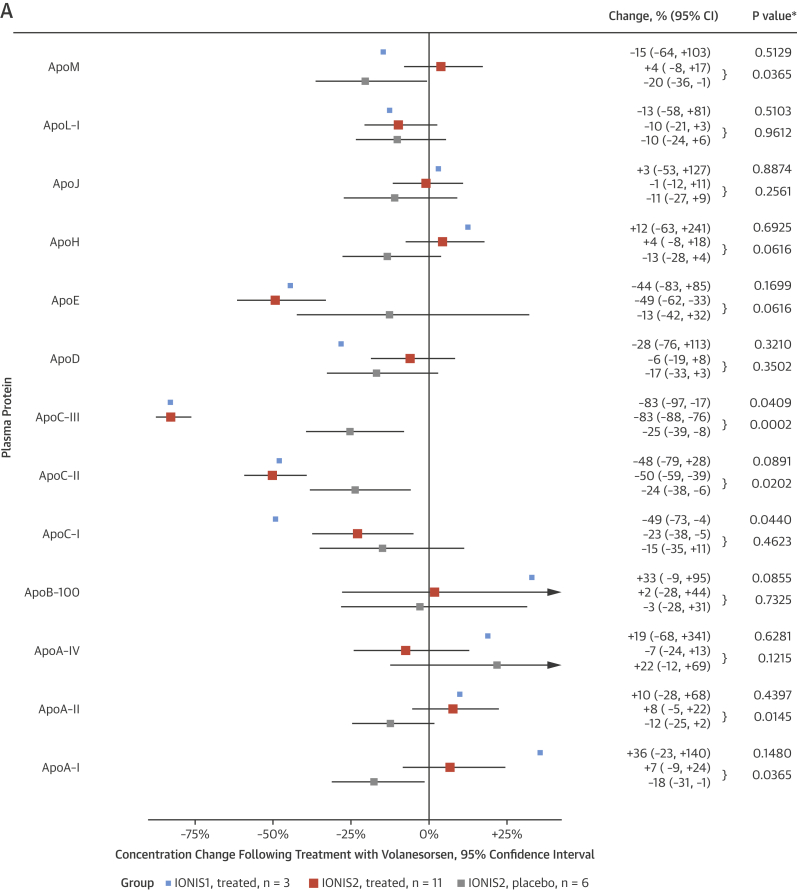

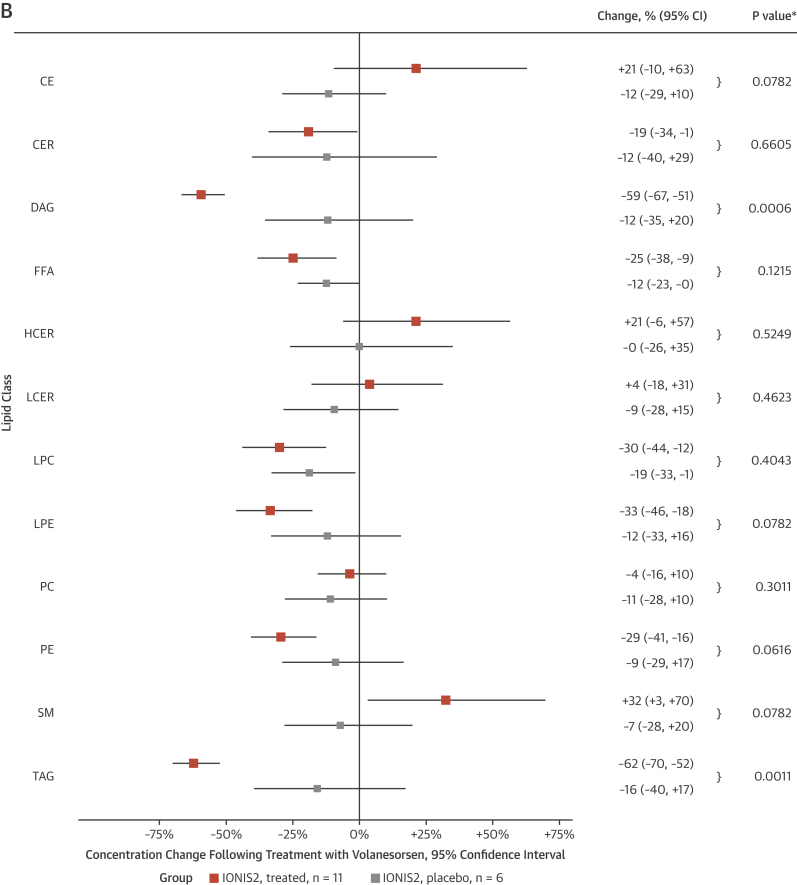
Effects of ApoC-III Synthesis Inhibition on Plasma Concentrations of Apolipoproteins and Lipid Classes In 2 independent intervention trials (IONIS1 and IONIS2), apoC-III synthesis was inhibited with the second-generation antisense oligonucleotide volanesorsen. In the randomized double-blind IONIS2 trial, 11 patients received treatment and 6 received placebo. *p values are for change from baseline in IONIS1, and for differential change from baseline in the treatment and placebo groups in IONIS2. **(A)** Effect on apolipoproteins. Among the apolipoproteins measured, apoC-III decreased most strongly, followed by apoC-II. An increase in plasma levels was observed for apoA-I, apoA-II, and apoM. **(B)** Effect on lipid classes. A substantial reduction in plasma levels was observed for TAG and DAG. CER = ceramide; DAG = diacylglycerol; FFA = free fatty acid; HCER = hexosylceramide; LCER = lactosylceramide; other abbreviations as in Figure 4.

In 2 recent clinical trials, a generation-2+ single-stranded antisense agent that targets hepatic *APOC3* messenger RNA, termed volanesorsen, was used to lower plasma apoC-III levels 2, 3. We obtained plasma samples from cohorts of these studies that had varying degrees of marked hypertriglyceridemia before and after volanesorsen therapy, and measured apolipoproteins and selected lipids by MRM-MS. As expected, inhibition of hepatic apoC-III synthesis substantially reduced plasma apoC-III levels in all subjects (mean decreases >75%). Remarkably, this was associated with ∼50% decreases in both apoC-II and apoE, and modest increases in apoA-I, apoA-II, and apoM, whereas levels of apoB-100 did not change, except in the 3 subjects with familial chylomicronemia syndrome (FCS) (IONIS1), who experienced a marked decrease in TGs (Figure 5A). Consistent with this, apoC-III inhibition lowered plasma concentrations of TAG as expected, but also lowered diacylglycerols (DAGs) (Figure 5B).

## Discussion

In an analysis of baseline samples from a prospective population-based study, apoC-III, apoC-II, and apoE were the apolipoproteins most strongly related to CVD (Central Illustration). ApoC-II, apoC-III, and apoE are abundant on TRLs, and profoundly modulate their metabolism (7). Our findings are consistent with a role of TRLs in the pathogenesis of CVD. The concept that TRL remnants penetrate the arterial intima where they promote atherosclerosis, similar to LDL 8, 9, is corroborated by recent Mendelian randomization studies causally implicating TRLs in CVD 10, 11, 12, 13, 14, 15.

### VLDL-associated apolipoproteins

Because samples were taken in the fasting state, the predominant TRL captured in our analysis is VLDL. After hepatic secretion, VLDL contains variable amounts of apoC-II/apoC-III/apoE, which in turn have variable and complex effects on the fate of the various VLDL constituents and on plasma TG levels. ApoE and apoC-III have been reported to stimulate hepatic secretion of VLDL in isolated hepatocyte cultures, but there is no evidence for such effects in vivo in humans 16, 17. However, apoE does play an important role in mediating rapid hepatic removal of TRLs by serving as a ligand to mediate binding to hepatic LDL receptors (LDLR) and LDL receptor-related protein 1 (LRP-1) (18). In rare patients who have complete apoE deficiency and/or in subjects homozygous for the E2/E2 apoE alleles, which have decreased affinity for LDLR, marked hypertriglyceridemia and even chylomicronemia can occur (19).

ApoC-II and apoC-III have opposing effects on plasma TG levels. Lipoprotein lipase (LPL) is primarily responsible for the hydrolysis of TGs in TRLs such as VLDL and chylomicrons. In the absence of LPL activity, as occurs in FCS, marked accumulation of both VLDL and chylomicrons occurs, resulting in massive hypertriglyceridemia, with TG values often exceeding 2,000 to 5,000 mg/dl, causing acute pancreatitis. ApoC-II is an obligate activator of LPL, and its absence can lead to FCS (20). Thus, apoC-II promotes VLDL-TG hydrolysis and the generation of smaller and denser VLDL remnants 18, 21. By contrast, apoC-III interferes with apoC-II–mediated activation of LPL, and thereby promotes hypertriglyceridemia (7). Indeed, it was previously thought that inhibiting LPL activity was the primary mechanism by which apoC-III raised plasma TG. However, the recent observation that lowering apoC-III levels in FCS patients by use of volanesorsen dramatically lowered patients’ plasma TG levels demonstrated conclusively that apoC-III also impaired TRL clearance by an LPL-independent pathway 2, 22, 23. This is thought to be due to inhibiting hepatic clearance of TRL lipoproteins mediated by LDLR or LRP-1 18, 21, 23. Thus, it is now apparent that apoC-III regulates TRL metabolism by both an LPL-dependent and LPL-independent pathway, and is thus a central regulator of plasma TG levels 2, 7, 18, 22.

ApoC-II, apoC-III, and apoE were associated with obesity, hypertension, impaired glucose metabolism, and most strongly with lipid parameters (Online Figure 4), particularly, CEs and TAGs with shorter and more saturated fatty acid chains (Online Figure 6). This pattern is consistent with hepatic de novo lipogenesis, and resembles the lipid signature of CVD previously observed in the Bruneck Study (1). Following adjustment for apoC-II/apoC-III/apoE, associations of lipid species with CVD were substantially attenuated, further emphasizing the relevance of TRLs for CVD risk.

### Triglycerides and apoC-III

Considerable epidemiological, genetic, and now therapeutic data have emerged to suggest that apoC-III is a central regulator of TRL metabolism 2, 3. It would be the logical preferred therapeutic target for lowering VLDL levels, as inhibiting hepatic VLDL release might theoretically lead to steatosis. Similarly, inhibition of apoE would have the net effect of reducing TRL clearance, and apoC-II is necessary for physiological VLDL and chylomicron TG lipolysis. Furthermore, genome-wide association and Mendelian randomization studies suggest that loss-of-function mutations of apoC-III confer cardiovascular protection 12, 13, 24, 25. The Framingham Study has linked apoC-III, as measured by immunoassays, to incident myocardial infarction or angina pectoris (12). We now provide the first data that apoC-III, along with apoC-II and apoE, associates significantly and independently with incident stroke and myocardial infarction on the basis of a direct comparison of apolipoprotein levels by MRM-MS, rather than immunoassays. ApoC-III in VLDL or LDL, as well as total plasma apoC-III, was associated with CVD, but results for HDL-bound apoC-III were ambiguous (26).

### Antisense inhibition of apoC-III

Antisense therapy that inhibits hepatic apoC-III synthesis results in effective reductions in plasma apoC-III and TG levels. Beneficial effects of apoC-III lowering may extend beyond the impact of lowering plasma TRL levels. ApoC-III loss of function also resulted in decreased LDL-C (12) and apoB-100 (13). Conversely, elevated apoC-III was linked to high levels of the particularly atherogenic small dense LDL (21), HDL dysfunction (27), and promotion of proteoglycan binding of LDL (28). Thus, we examined the effects of apoC-III synthesis inhibition by volanesorsen on apolipoprotein levels in 2 human intervention trials, 1 in FCS subjects (IONIS1), who lack LPL activity (2), and 1 in subjects with prominent hypertriglyceridemia (IONIS2) of varied etiology (Figure 5A) (3). The reduction in apoC-III levels was profound, leading to >75% decreases at the dose of antisense used. This was associated not only with marked reductions in plasma TGs of ∼70%, but there were nearly 50% decreases in both apoC-II and apoE. These changes are consistent with lowering of VLDL and remnant lipoproteins 7, 21, and are in line with observations after apoC-III inhibition in mice, nonhuman primates, and humans 2, 3, 29. However, the disparity between the extent of reduction in apoC-III, apoC-II, and apoE suggests some measures of independence in the metabolism of these 3 apolipoproteins, and it is well known that they may reside on other lipoproteins, such as HDL (30). Indeed, the observed increase in apoA-I, apoA-II, and apoM is consistent with the reported rise of HDL-C following apoC-III inhibition 3, 29 and lower HDL-C levels in apoC-III transgenic mice (31). A potential mechanistic explanation is reduced exchange of HDL-C with VLDL-TG mediated via cholesterol ester transfer protein (CETP) 7, 29. Notably, apoM has been reported to mark an HDL subpopulation that stimulates particularly efficient cholesterol efflux (32).

Although apoC-III delays clearance of VLDL remnants that contain apoB-100, apoC-III inhibition by volanesorsen did not reduce total apoB-100 levels. This may be explained by the fact that although VLDL-apoB levels were decreased 2, 3, there was a small compensatory increase in LDL. This was likely in part due to CETP-mediated remodeling of the lipoprotein cholesterol content (3), increased conversion of VLDL to LDL mediated by LPL, or changes in so-called metabolic channeling of VLDL to small dense LDL 7, 14, 15, 18. Importantly, total apoB levels did not increase or were slightly decreased in the volanesorsen-treated hypertriglyceridemic subjects who were on other hypolipidemic agents (3). This would explain why only 16% lower apoB-100 levels were reported in carriers of apoC-III loss-of-function mutations with normal TG levels (13), although a recent report of such subjects did not find lower LDL levels (25). Following apoC-III inhibition, TAGs and DAGs were decreased (Figure 5B). Although the former is expected, the latter is notable because DAGs are precursors of TAGs in the last step of TG synthesis (33). Inhibition of hepatic TG synthesis might thus be relevant for the TAG-lowering effects upon apoC-III inhibition, although changes in VLDL-TG secretion were not seen in volanesorsen-treated apoC-III transgenic mice (29). However, lipid metabolism in mice is different from that in humans. For example, mice do not have plasma activity of the CETP that facilitates the exchange of CE for TG between HDL and TG-rich remnant lipoproteins. As a consequence, HDL is the major cholesterol carrying lipoprotein in mice, but not in humans.

The observational and interventional results presented in this study suggest that TG levels are a modifiable CVD risk factor. Although randomized trials testing TG lowering for CVD prevention have reported mixed results (8), a meta-regression of the results shows a dose–effect relation between degree of TG lowering and CVD risk reduction (8). For subjects with high TG levels, this relation was accentuated, and the results in individual studies were also consistently significant (8). Thus, the present study is in line with prior reports suggesting TG lowering as a potential therapeutic approach.

### Study strengths

Strengths of this study include its representativeness for the general population, rigorous endpoint evaluation, near-complete follow-up, and comparability of observational and interventional data, facilitated by identical protein measurement methods using MRM-MS, rather than immunoassays. Variability in antibody sensitivity and specificity can hamper direct comparisons of biomarkers using antibody-based assays. At present, MRM-MS is not a high-throughput method, but has the advantage of measuring proteins directly, without a binder. The range of apolipoproteins investigated by MRM-MS for association with incident CVD in a population-based cohort is unprecedented.

### Study limitations

Weaknesses of this study include that the many correlated tests presented herein were not adjusted for multiplicity, although key results would resist such adjustment, and that use of statins may weaken the association of VLDL- and LDL-associated apolipoproteins with regard to CVD risk; however, <10% of participants in the Bruneck study were on statin therapy, and exclusion of subjects on statin therapy yielded similar results (Online Figure 3). Future studies could extend the present study by measuring apolipoproteins within lipoprotein subfractions. Our findings of strong associations with apolipoproteins, such as apoL-I and apoH, for which published data are lacking, should be considered hypothesis-generating and deserving of further study.

## Conclusions

Our data provide strong epidemiological support to the concept that TRLs contribute to atherosclerosis. ApoC-II, apoC-III, and apoE are abundant on VLDL, which may represent an underappreciated risk factor for CVD. The interventional trials with volanesorsen demonstrate that targeting apoC-III favorably affects apolipoprotein and lipid profiles. Thus, lowering VLDL, in addition to LDL and lipoprotein(a), might represent a novel strategy to further reduce CVD risk in the statin era, and could be tested by appropriately designed outcomes trials.

Perspectives **COMPETENCY IN MEDICAL KNOWLEDGE:** The apolipoproteins apoC-III, apoC-II, and apoE are found on triglyceride-rich lipoproteins, regulate their metabolism, and outperform other apolipoproteins, including apoB-100 and apoA-I, as predictors of cardiovascular events.

**TRANSLATIONAL OUTLOOK:** Further studies are needed to assess the impact of inhibiting apoC-III synthesis on clinical cardiovascular outcomes.

## Figures and Tables

**Central Illustration:**
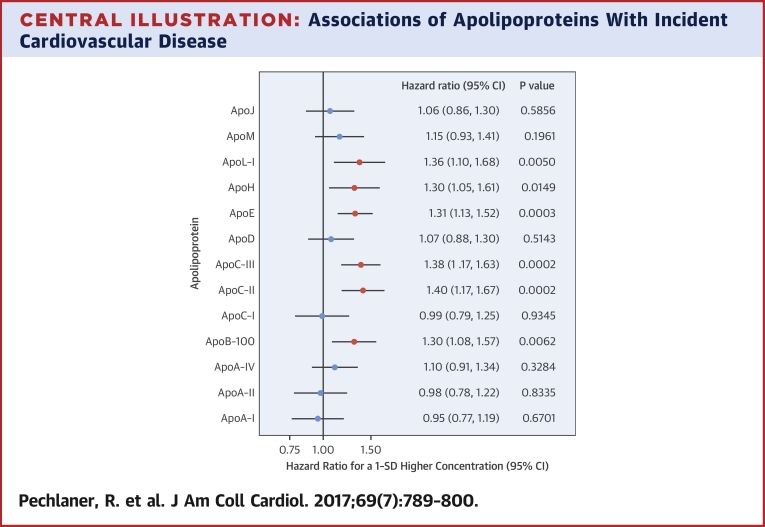
Associations of Apolipoproteins With Incident Cardiovascular Disease Plasma levels of 13 apolipoproteins were determined in 688 participants of the Bruneck Study. During 10 years of follow-up, 91 cardiovascular events occurred, comprising stroke, myocardial infarction, and sudden cardiac death. Results are adjusted for age, sex, and statin therapy. Apo = apolipoprotein; CI = confidence interval.

## References

[1] Stegemann C., Pechlaner R., Willeit P. (2014). Lipidomics profiling and risk of cardiovascular disease in the prospective population-based Bruneck Study. Circulation.

[2] Gaudet D., Brisson D., Tremblay K. (2014). Targeting APOC3 in the familial chylomicronemia syndrome. N Engl J Med.

[3] Gaudet D., Alexander V.J., Baker B.F. (2015). Antisense inhibition of apolipoprotein C-III in patients with hypertriglyceridemia. N Engl J Med.

[4] (1971). IHD Register: Report of the Fifth Working Group.

[5] Walker A.E., Robins M., Weinfeld F.D. (1981). The National Survey of Stroke: clinical findings. Stroke.

[6] Rothman K.J. (1990). No adjustments are needed for multiple comparisons. Epidemiology (Cambridge, Mass).

[7] Jong M.C., Hofker M.H., Havekes L.M. (1999). Role of ApoCs in lipoprotein metabolism functional differences between ApoC1, ApoC2, and ApoC3. Arterioscler Thromb Vasc Biol.

[8] Nordestgaard B.G., Varbo A. (2014). Triglycerides and cardiovascular disease. Lancet.

[9] Rapp J.H., Harris H.W., Hamilton R.L. (1989). Particle size distribution of lipoproteins from human atherosclerotic plaque: a preliminary report. J Vasc Surg.

[10] Do R., Willer C.J., Schmidt E.M. (2013). Common variants associated with plasma triglycerides and risk for coronary artery disease. Nat Genet.

[11] Jørgensen A.B., Frikke-Schmidt R., West A.S. (2013). Genetically elevated non-fasting triglycerides and calculated remnant cholesterol as causal risk factors for myocardial infarction. Eur Heart J.

[12] TG and HDL Working Group of the Exome Sequencing Project, National Heart, Lung, and Blood Institute (2014). Loss-of-function mutations in *APOC3*, triglycerides, and coronary disease. N Engl J Med.

[13] Jørgensen A.B., Frikke-Schmidt R., Nordestgaard B.G. (2014). Loss-of-function mutations in APOC3 and risk of ischemic vascular disease. N Engl J Med.

[14] Varbo A., Benn M., Tybjærg-Hansen A. (2013). Remnant cholesterol as a causal risk factor for ischemic heart disease. J Am Coll Cardiol.

[15] Varbo A., Benn M., Tybjærg-Hansen A. (2013). Elevated remnant cholesterol causes both low-grade inflammation and ischemic heart disease, whereas elevated low-density lipoprotein cholesterol causes ischemic heart disease without inflammation. Circulation.

[16] Sundaram M., Zhong S., Bou Khalil M. (2010). Expression of apolipoprotein C-III in McA-RH7777 cells enhances VLDL assembly and secretion under lipid-rich conditions. J Lipid Res.

[17] Mensenkamp A.R., Jong M.C., van Goor H. (1999). Apolipoprotein E participates in the regulation of very low density lipoprotein-triglyceride secretion by the liver. J Biol Chem.

[18] Sacks F.M. (2015). The crucial roles of apolipoproteins E and C-III in apoB lipoprotein metabolism in normolipidemia and hypertriglyceridemia. Curr Opin Lipidol.

[19] Gabelli C., Gregg R.E., Zech L.A. (1986). Abnormal low density lipoprotein metabolism in apolipoprotein E deficiency. J Lipid Res.

[20] Mahley R.W., Huang Y., Rall S.C. (1999). Pathogenesis of type III hyperlipoproteinemia (dysbetalipoproteinemia): questions, quandaries, and paradoxes. J Lipid Res.

[21] Mendivil C.O., Zheng C., Furtado J. (2010). Metabolism of very-low-density lipoprotein and low-density lipoprotein containing apolipoprotein C-III and not other small apolipoproteins. Arterioscler Thromb Vasc Biol.

[22] Drenos F., Davey Smith G., Ala-Korpela M. (2016). Metabolic characterization of a rare genetic variation within APOC3 and its lipoprotein lipase-independent effects. Circ Cardiovasc Genet.

[23] Gordts P.L., Nock R., Son N.H. (2016). ApoC-III inhibits clearance of triglyceride-rich lipoproteins through LDL family receptors. J Clin Invest.

[24] Pollin T.I., Damcott C.M., Shen H. (2008). A null mutation in human APOC3 confers a favorable plasma lipid profile and apparent cardioprotection. Science.

[25] Natarajan P., Kohli P., Baber U. (2015). Association of APOC3 loss-of-function mutations with plasma lipids and subclinical atherosclerosis: the multi-ethnic BioImage study. J Am Coll Cardiol.

[26] Wyler von Ballmoos M.C., Haring B., Sacks F.M. (2015). The risk of cardiovascular events with increased apolipoprotein CIII: A systematic review and meta-analysis. J Clin Lipidol.

[27] Riwanto M., Rohrer L., Roschitzki B. (2013). Altered activation of endothelial anti- and proapoptotic pathways by high-density lipoprotein from patients with coronary artery disease role of high-density lipoprotein–proteome remodeling. Circulation.

[28] Hiukka A., Ståhlman M., Pettersson C. (2009). ApoCIII-enriched LDL in type 2 diabetes displays altered lipid composition, increased susceptibility for sphingomyelinase, and increased binding to biglycan. Diabetes.

[29] Graham M.J., Lee R.G., Bell T.A. (2013). Antisense oligonucleotide inhibition of apolipoprotein C-III reduces plasma triglycerides in rodents, nonhuman primates, and humans. Circ Res.

[30] Yang X., Lee S.R., Choi Y.S. (2016). Reduction in lipoprotein-associated apoC-III levels following volanesorsen therapy: phase 2 randomized trial results. J Lipid Res.

[31] Han Y., Qi R., Liu G. (2015). Reduced high density lipoprotein cholesterol in severe hypertriglyceridemic ApoCIII transgenic mice via lowered hepatic ApoAI synthesis. Biochem Biophys Res Commun.

[32] Christoffersen C., Nielsen L.B., Axler O. (2006). Isolation and characterization of human apolipoprotein M-containing lipoproteins. J Lipid Res.

[33] Coleman R.A., Lee D.P. (2004). Enzymes of triacylglycerol synthesis and their regulation. Prog Lipid Res.

