# Supplementary material for: Very-Low-Density Lipoprotein–Associated Apolipoproteins Predict Cardiovascular Events and Are Lowered by Inhibition of APOC-III
Source: J Am Coll Cardiol. 2017 Feb 21;69(7):789–800. doi: 10.1016/j.jacc.2016.11.065 (PMC5314136; doi:10.1016/j.jacc.2016.11.065)

Online Appendix to:

VERY LOW-DENSITY LIPOPROTEIN ASSOCIATED APOLIPOPROTEINS PREDICT CARDIOVASCULAR EVENTS AND ARE LOWERED BY INHIBITION OF APOC-III

Raimund Pechlaner[a], MD, PhD, Sotirios Tsimikas[b,c], MD, Xiaoke Yin[d], PhD, Peter Willeit[a,e], MD, PhD, Ferheen Baig[d], MSc, Peter Santer[f], MD, Friedrich Oberhollenzer[f], MD, Georg Egger[g], MD, Joseph L. Witztum[b], MD, Veronica J. Alexander[c], PhD, Johann Willeit[a], MD, Stefan Kiechl[a], MD, Manuel Mayr[d], MD, PhD

[a]Department of Neurology, Medical University Innsbruck, Anichstraße 35, Innsbruck, Austria;
[b]University of California San Diego, 9500 Gilman Dr, La Jolla, CA, USA;
[c]Ionis Pharmaceuticals, 2855 Gazelle Ct, Carlsbad, CA, USA;
[d]King's British Heart Foundation Centre, 125 Coldharbour Lane, King's College London, London, UK,
[e]Department of Public Health and Primary Care, University of Cambridge, 2 Worts' Causeway, Cambridge, UK;
[f]Department of Laboratory Medicine, Hospital of Bruneck, Spitalstraße 11, Bruneck, Italy;
[g]Department of Internal Medicine, Hospital of Bruneck, Spitalstraße 11, Bruneck, Italy.

Address for Correspondence:

Manuel Mayr; King's British Heart Foundation Centre, King's College London, 125 Coldharbour Lane, London, SE59NU UK; Phone: +44 (0) 20 7848 5446; +44 (0) 20 7848 5298; Email: manuel.mayr@kcl.ac.uk

or

Stefan Kiechl; Department of Neurology, Medical University Innsbruck, Anichstraße 35, 6020 Innsbruck, Austria; Phone: +43 (0) 512 504 24244; Fax: +43 (0) 512 504 23987; Email: stefan.kiechl@i-med.ac.at

ONLINE METHODS

The Bruneck Study. The Bruneck Study is a prospective, population-based study with a prime focus on atherosclerosis and CVD and related risk factors (1-3). In 1990, 1,000 individuals were recruited as a random sample of Bruneck inhabitants and re-examined every 5 years (participation >90% each). The present study used the 2000 survey as baseline. Full medical records were available on all clinical endpoints occurring between 2000 and 2010 for all 688 individuals (100% follow-up). CVD was defined as myocardial infarction, stroke, or sudden cardiac death. Fatal and nonfatal myocardial infarction was deemed confirmed when World Health Organization criteria for definite disease status were met. Ischemic stroke was classified according to the criteria of the National Survey of Stroke. The Bruneck Study protocol conformed to the Declaration of Helsinki and was approved by the ethics committees of Bolzano and Verona. All study participants gave their written informed consent. Risk factors were ascertained by validated standard procedures as previously described (1,4,5). Blood samples were taken after an overnight fast. Lipidomic profiling was performed with MS, which allowed quantification of 135 distinct lipid species (1). HbA1c was quantified using high performance liquid chromatography (DCCT-aligned assay).

Volanesorsen phase 2 trials. Plasma samples collected in EDTA were available from two recently described phase 2 trials (6,7). They were taken prior to drug administration and at days 57 and 92 (end of treatment) following initiation of volanesorsen (previously known as ISIS 304801 and ISIS-APOCIII$_{Rx,}$ by Ionis Pharmaceuticals, formerly Isis Pharmaceuticals) treatment. In the first study (IONIS1) 3 patients with familial chylomicronemia syndrome were treated with volanesorsen in an open label study (6). The second study (IONIS2) (7) was a phase 2, randomized, double-blind, placebo-controlled, dose-ranging trial of subjects with hypertriglyceridemia, and we here studied the subset of 17 patients without current treatment with other lipid lowering agents (11 volanesorsen and 6 placebo). Both studies used the same protocol with subcutaneous, once weekly injections of 300 mg volanesorsen for 13 weeks. Written informed consent was received from participants prior to inclusion in the studies. The study protocol of this trial is available online (7). Participants of the IONIS2 trial in the volanesorsen monotherapy arm (7) were considered eligible if they had fasting triglyceride levels between 350 mg per deciliter (4.0 mmol per liter) and 2000 mg per deciliter. They were initially randomly assigned in a 3:1 ratio to receive active agent (100mg, 200mg, or 300mg) or placebo. For the current study, only samples from participants who received 300mg or the corresponding placebo participants were available. The placebo patients were dosed according to the same timetable and with the same injection volume as the treated patients. The unblinded pharmacist (or qualified delegate) preparing the dosing solutions was the only member of study staff to know which patient was receiving placebo.

Proximity extension assay. Proximity Extension Assay (PEA) technology, using Proseek Multiplex CVD I 96x96 and Proseek Multiplex Inflammation I 96x96 reagents kits (Olink Bioscience, Uppsala, Sweden), was employed to measure low-abundant plasma proteins. Each assay measured 92 proteins. We excluded 29 proteins with >25% of values below detection limit and 23 that were measured in both panels, leaving 132 individual proteins.

Lipidomics. Lipids in the Bruneck Study were analyzed by MS in the presence of authentic internal standards. For the IONIS2 study, lipidomic analysis was performed using the TrueMass Complex Lipid Panel (Metabolon) combined with SelexIon differential mobility spectroscopy (Sciex). Lipids were extracted from samples in methanol:dichloromethane in the presence of internal standards. The extracts were concentrated under nitrogen and reconstituted in 0.25mL of 10mM ammonium acetate dichloromethane:methanol (50:50). The extracts were transferred to inserts and placed in vials for infusion-MS analysis, performed on a Shimazdu LC with nano PEEk tubing and the Sciex SelexIon-5500 QTRAP. The samples were analyzed via both positive and negative mode electrospray. The 5500 QTRAP scan was performed in MRM mode with the total of more than 1,100 MRMs. Individual lipid species were quantified by taking the peak area ratios of target compounds and their assigned internal standards, then multiplying by the concentration of internal standard added to the sample. Lipid class concentrations were calculated from the sum of all molecular species within a class, and fatty acid compositions are determined by calculating the proportion of each class comprised by individual fatty acids.

MiR-122 measurement. We measured miRNA-122 in plasma samples of the Bruneck Study using real time PCR (qRT-PCR), as previously described (8,9). Briefly, miRNAs were extracted using the miRNeasy kit (Qiagen, Hilden, Germany). For plasma, a fixed volume of 3µl of the 25µl RNA eluate was used as input for reverse transcription (RT) reactions. MiRNAs were reversely transcribed using Megaplex Primer Pools (Human Pool A version 2.1 or Rodent Pool A, Life Technologies, Darmstadt, Germany) and products were further amplified using Megaplex PreAmp Primers (Primers A v2.1). Both RT and PreAmp products were stored at -20°C. Taqman miRNA assays were used to assess the expression of individual miRNAs. Diluted pre-amplification product (0.5µl) or RT product (corresponding to 0.45ng input) were combined with 0.25µl Taqman microRNA assay (20×) (Life Technologies) and 2.5µl Taqman Universal PCR Master Mix No AmpErase UNG (2×) to a final volume of 5µl. qRT-PCR was performed on an Applied Biosystems 7900HT thermocycler at 95°C for 10min, followed by 40 cycles of 95°C for 15s and 60°C for 1min. All samples were run in duplicate. Laboratory technicians were blinded to the participants' disease status. Relative quantification was performed using the

software SDS2.2 (Life Technologies). Exogenous *C. elegans* spike-in control (*Cel-miR-39*) was used for normalisation purposes.

Statistical methods for risk prediction analyses. We assessed the predictive value added by the apolipoproteins most strongly associated with CVD, apoC-III, apoC-II, and apoE, when added to a model that already included the traditional risk factors age, sex, systolic blood pressure, smoking, diabetes, and also statin therapy. Model discrimination, that is, the ability of the model to discern events from non-events, was gauged using the difference in Harrell's c-index between these models. The c-index is a generalization of the area under the receiver operator curve suitable for time-to-event data (10) and can be interpreted as the probability that the model will correctly predict the order of event occurrence among a random pair of individuals for which such ordering is possible. Perfect discrimination is indicated by a value of 1, while a value of 0.5 is expected by chance.

Discrimination was further investigated using the integrated discrimination improvement (IDI) and its "relative" variant (11,12). Both variants of the IDI first estimate the difference in average predicted risks for subjects that experience events and for subjects that do not for both the new and the old model. The IDI estimates the absolute change in these risk differences upon additionally considering apolipoproteins (difference in discrimination slopes), while the relative IDI estimates the proportional change.

Improvement in model calibration, that is, the ability of a model to accurately estimate risk, was assessed using the prospective formulation of the net reclassification improvement (NRI) (13) using risk categories formed by cut-offs of 5% and 7.5% 10-year CVD risk, and using the continuous variant of the NRI (13). The former assessed the degree to which subjects could be more appropriately classified into the clinically relevant risk categories of 0.0-5.0%, 5.0-7.5%, and 7.5%-100% 10-year CVD risk upon additional consideration of the three apolipoproteins, while the latter investigated for what proportion of subjects risk was predicted more appropriately by the new model.

The assessment of incremental predictive value was implemented within a 10-fold cross-validation scheme (10 000 repetitions) such that models were trained on data separate from the data they were assessed on. The aim of this procedure was to estimate expected out-of-sample predictive performance. Confidence intervals for statistics indicating predictive performance were calculated as bootstrap percentile confidence intervals (1000 repetitions) within the cross-validation scheme (nested resampling).

ONLINE REFERENCES

1. Stegemann C, Pechlaner R, Willeit P et al. Lipidomics profiling and risk of cardiovascular disease in the prospective population-based Bruneck Study. Circulation 2014;129:1821-1831.
2. Kiechl S, Lorenz E, Reindl M et al. Toll-like receptor 4 polymorphisms and atherogenesis. N Engl J Med 2002;347:185-192.
3. Kiechl S, Wittmann J, Giaccari A et al. Blockade of receptor activator of nuclear factor-κB (RANKL) signaling improves hepatic insulin resistance and prevents development of diabetes mellitus. Nat Med 2013;19:358-363.
4. Pechlaner R, Willeit P, Summerer M et al. Heme oxygenase-1 gene promoter microsatellite polymorphism is associated with progressive atherosclerosis and incident cardiovascular disease. Arterioscler Thromb Vasc Biol 2015;35:229-236.
5. Pechlaner R, Kiechl S, Mayr M et al. Correlates of serum hepcidin levels and its association with cardiovascular disease in an elderly general population. Clin Chem Lab Med 2016 ;54:151-61.
6. Gaudet D, Brisson D, Tremblay K et al. Targeting APOC3 in the familial chylomicronemia syndrome. N Engl J Med 2014;371:2200-2206.
7. Gaudet D, Alexander VJ, Baker BF et al. Antisense inhibition of apolipoprotein C-III in patients with hypertriglyceridemia. N Engl J Med 2015;373:438-447.
8. Zampetaki A, Willeit P, Tilling L et al. Prospective study on circulating microRNAs and risk of myocardial infarction. J Am Coll Cardiol 2012;60:290-299.
9. Willeit P, Zampetaki A, Dudek K et al. Circulating microRNAs as novel biomarkers for platelet activation. Circ Res 2013;112:595-600.
10. Pencina MJ, D'Agostino RB. Overall C as a measure of discrimination in survival analysis: model specific population value and confidence interval estimation. Stat Med 2004;23:2109–2123.
11. Pencina MJ, D' Agostino RB, D' Agostino RB et al. Evaluating the added predictive ability of a new marker: From area under the ROC curve to reclassification and beyond. Stat Med 2008;27:157–172.
12. Pencina MJ, D'Agostino RB, D'Agostino RB et al. Comments on 'Integrated discrimination and net reclassification improvements—Practical advice'. Stat Med 2008;27:207–212.
13. Pencina MJ, D'Agostino RB, Steyerberg EW. Extensions of net reclassification improvement calculations to measure usefulness of new biomarkers. Stat Med 2011;30:11–21.

ONLINE FIGURES

Online Figure 1:

Associations of Apolipoproteins and Lipids with Incident Stroke and Myocardial Infarction.

Plasma concentrations of 13 apolipoproteins and of 4 traditional lipid measures were measured in 688 participants of the Bruneck Study. SD, standard deviation

- **a)** During 10 years of follow-up, 50 strokes occurred.
- **b)** During 10 years of follow-up, 40 myocardial infarctions occurred.

Model 1: Adjustment for age, sex, and statin therapy.

Model 2: As model 1, with additional adjustment for diabetes, systolic blood pressure, and smoking.

Model 3: As model 2, with additional adjustment for HDL cholesterol and non-HDL cholesterol.

Online Figure 2:

Associations of Apolipoproteins and Lipid Measures with Incident CVD in Subjects without Prior Cardiovascular Disease.

Plasma levels of 13 apolipoproteins and of 4 conventional lipid measures were determined in 644 participants of the Bruneck Study who had not suffered cardiovascular events prior to baseline. During 10 years of follow-up, 76 cardiovascular events occurred, comprising stroke, myocardial infarction, and sudden cardiac death. SD, standard deviation.

Model 1: Adjustment for age, sex, and statin therapy.

Model 2: As in model 1, with additional adjustment for diabetes, systolic blood pressure, and smoking.

Model 3: As in model 2, with additional adjustment for HDL cholesterol and non-HDL cholesterol.

Quantitatively, for each variable one standard deviation corresponds to: ApoA-I, 607 mg/L; ApoA-II, 6.44 mg/L; ApoA-IV, 15.0 mg/L; ApoB-100, 363 mg/L; ApoC-I, 6.46 mg/L; ApoC-II, 6.30 mg/L; ApoC-III, 25.6 mg/L; ApoD, 7.98 mg/L; ApoE, 9.23 mg/L; ApoH, 38.2 mg/L; ApoL-I, 3.93 mg/L; ApoM, 2.42 mg/L; ApoJ, 23.1 mg/L; HDL-C, 15.2 mg/dL; LDL-C, 36.5 mg/dL; Non-HDL-C, 41.4 mg/dL; Triglycerides, 77.6 mg/dL;

Online Figure 3:

Associations of Apolipoproteins and Lipid Measures with Incident CVD in Subjects without Statin Therapy.

Plasma levels of 13 apolipoproteins and of 4 conventional lipid measures were determined in 624 participants of the Bruneck Study who did not receive statin therapy at baseline. During 10 years of follow-up, 78 cardiovascular events occurred, comprising stroke, myocardial infarction, and sudden cardiac death. SD, standard deviation.

Model 1: Adjustment for age and sex.

Model 2: As in model 1, with additional adjustment for diabetes, systolic blood pressure, and smoking.

Model 3: As in model 2, with additional adjustment for HDL cholesterol and non-HDL cholesterol.

Quantitatively, for each variable one standard deviation corresponds to: ApoA-I, 607 mg/L; ApoA-II, 6.44 mg/L; ApoA-IV, 15.0 mg/L; ApoB-100, 363 mg/L; ApoC-I, 6.46 mg/L; ApoC-II, 6.30 mg/L; ApoC-III, 25.6 mg/L; ApoD, 7.98 mg/L; ApoE, 9.23 mg/L; ApoH, 38.2 mg/L; ApoL-I, 3.93 mg/L; ApoM, 2.42 mg/L; ApoJ, 23.1 mg/L; HDL-C, 15.2 mg/dL; LDL-C, 36.5 mg/dL; Non-HDL-C, 41.4 mg/dL; Triglycerides, 77.6 mg/dL;

Online Figure 4:

Associations of ApoC-III, ApoC-II, and ApoE with Demographic, Metabolic, and Other Variables.

The 3 apolipoproteins showed similarly direct relations to body mass index and waist-hip ratio, alcohol consumption, liver enzymes, and systolic blood pressure, fasting glucose, and HbA1c. Relations to blood lipids also tended to be similar but were most pronounced for apoC-II and apoC-III, in particular for triglycerides, and only apoC-II was inversely related to HDL cholesterol. Results are derived from linear regression and are adjusted for age, sex, and statin therapy. For variables that were analysed on the log scale, geometric mean and geometric standard deviation are presented, for female sex the proportion of females.

Online Figure 5:

Associations of (a) ApoC-II, (b) ApoC-III, and (c) ApoE with Plasma Proteins.

Age-, sex-, and statin therapy-adjusted Pearson correlation coefficients are shown for the pairwise associations between apoC-II, apoC-III, and apoE with 224 plasma proteins. Positive correlations are coded as solid horizontal lines, negative correlations, dashed. Proteins are annotated and color-coded based on the biological process their primary function best relates to, and proteins with rare or unknown function are not annotated and colored grey.

Proteins that correlated with miR-122 at a q<0.05 level are marked with filled circles, others, with hollow circles. Only correlations significant at an FDR q<0.05 are shown and proteins with a skewness>2 were log-transformed before analysis. The following proteins were considered:

High abundant proteins:

92 high abundant plasma proteins measured by the Biognosys PlasmaDive MRM-MS assay

Low abundant proteins:

64 low abundant proteins measured by the Proseek Multiplex CVD I proximity extension assay

Low abundant proteins:

68 low abundant proteins measured by the Proseek Multiplex Inflammation I proximity extension assay;

Certain proteins were part of both Proseek Multiplex assays (n=23) and the measurements of the Inflammation panel were used for further analysis. A total of 52 proteins were not included in this analysis, i.e. the following proteins in the Proseek Multiplex CVD I panel had more than 25% values below detection limit (n=5): Pentraxin-related protein PTX3 (PTX3), Membrane-bound aminopeptidase P (mAmP), Melusin (ITGB1BP2), Interleukin-4 (IL-4), Natriuretic peptides B (BNP). The following proteins in the Proseek Multiplex Inflammation panel had more than 25% values below detection limit (n=24) and were not used: Interleukin-17A (IL-17A), Interleukin-20 receptor subunit alpha (IL-20RA), Interleukin-2 receptor subunit beta (IL-2RB), Interleukin-1 alpha (IL-1 alpha), Interleukin-2 (IL-2), Thymic stromal lymphopoietin (TSLP), Interleukin-10 receptor subunit alpha (IL-10RA), Fibroblast growth factor 5 (FGF-5), Matrix metalloproteinase-1 (MMP-1), Interleukin-15 receptor subunit alpha (IL-15RA), Interleukin-22 receptor subunit alpha-1 (IL-22 RA1), Programmed cell death 1 ligand 1 (PD-L1), Beta-nerve growth factor (Beta-NGF), Interleukin-24 (IL-24), Interleukin-13 (IL-13), Artemin (ARTN), Tumor necrosis factor (TNF), Interleukin-20 (IL-20), Interleukin-33 (IL-33), Interferon gamma (IFN-gamma), Interleukin-4, (IL-4), Leukemia inhibitory factor (LIF), Neurturin (NRTN), Interleukin-5 (IL-5).

Online Figure 6:

Associations of Molecular Lipid Species with a) ApoC-II, b) ApoC-III and c) ApoE, with CVD, and with CVD under Adjustment for these Apolipoproteins.

Individual molecular lipid species are grouped in panel columns by their class, which is determined by their characteristic head group. They are arranged according to their fatty acid chain length (number of carbon atoms)

along the x-axis, and according to their unsaturation (double bond content) along the y-axis. Head group and fatty acid chains are linked by either an ether linkage or an ester linkage, and lipid species possessing at least one ether linkage are circled in grey, while species only possessing ester linkages are circled in black. Strength and direction of the association are represented as circle fill colour hue, significance level of association as circle size. Results are adjusted for age, sex, and statin therapy.

The *top row* shows SD unit increases in lipid species concentrations associated with a one-SD higher apolipoprotein concentration.
The *middle row* shows log hazard ratios for CVD associated with a one-SD higher lipid species concentration.
The *bottom row* shows log hazard ratios for CVD associated with a one-SD higher lipid species concentration, when extending multivariable adjustment for apolipoprotein.

CE indicates cholesteryl ester; LPC, lysophosphatidylcholine; LPE, lysophosphatidylethanolamine; PC, phosphatidylcholine; PE, phosphatidylethanolamine; PS, phosphatidylserine; SM, sphingomyelin; and TAG, triacylglycerol.

For example in Online Figure 6B, TAG(54:2), shown in the rightmost column, is a TAG species in total possessing 54 carbon atoms (x axis) and 2 double bonds (y axis) in its fatty acid chains, which are bound to the glycerol head group by ester linkages (black circle outline). It is strongly and highly significantly associated with both apoC-III ($P<0.001$, top panel) and with CVD ($P<0.001$, middle panel), and upon extending multivariable adjustment for apoC-III its association with CVD is markedly attenuated ($P<0.05$, bottom panel).

<u>Online Table 1:</u> Baseline Characteristics.

Values are given as count (percentage), as mean ± standard deviation, or as median (interquartile range).

**Variable**	**Value**
Female sex, n (%)	357 (51.9)
Age, years	66.0 ± 10.2
Current smoking, n (%)	112 (16.4)
Total cholesterol, mg/dL	233.3 ± 41.9
HDL cholesterol, mg/dL	57.2 ± 15.2
Non-HDL cholesterol, mg/dL	176.0 ± 41.4
LDL cholesterol, mg/dL	148.3 ± 36.8
Triglycerides, mg/dL	137.1 ± 77.6
Statin therapy, n (%)	64 (9.3)
Systolic blood pressure, mmHg	139.7 ± 18.7
Diabetes mellitus, n (%)	74 (10.8)
Prior CVD, n (%)	44 (6.4)

<u>Online Table 2:</u> Incremental Predictive Value Afforded by ApoC-III, ApoC-II, and ApoE.

Results compare a base model including age, sex, statin therapy, smoking, diabetes, and systolic blood pressure to a model additionally including apoC-III, apoC-II, and apoE. Individual c-indices for these models (95% confidence intervals) were 0.704 (0.652, 0.755) and 0.723 (0.673, 0.770), respectively.

Results are based on a 10-fold cross-validation scheme with 10 000 repetitions, and confidence intervals are bootstrap percentile confidence intervals based on 1000 repetitions. Confidence intervals were cross-validated in the same way the other statistics were.

NRI is based on 10-year risk cut-offs of 5% and 7.5%.

There occurred 91 cardiovascular events in 688 subjects during 10 years of follow-up.

NRI, net reclassification index; IDI, integrated discrimination improvement;

**Statistic**		**Estimate**	**95% confidence limits**	
			**Lower**	**Upper**
**Δc-index**		0.018	-0.005	0.043
**NRI**	**Total**	0.123	0.047	0.201
	**Events**	0.022	-0.044	0.090
	**Non-events**	0.101	0.063	0.139
**Continuous NRI**	**Total**	0.323	0.111	0.537
	**Events**	-0.038	-0.235	0.160
	**Non-events**	0.362	0.282	0.440
**IDI**		0.017	0.000	0.036
**Relative IDI**		0.195	0.001	0.474

Online Figure 1A

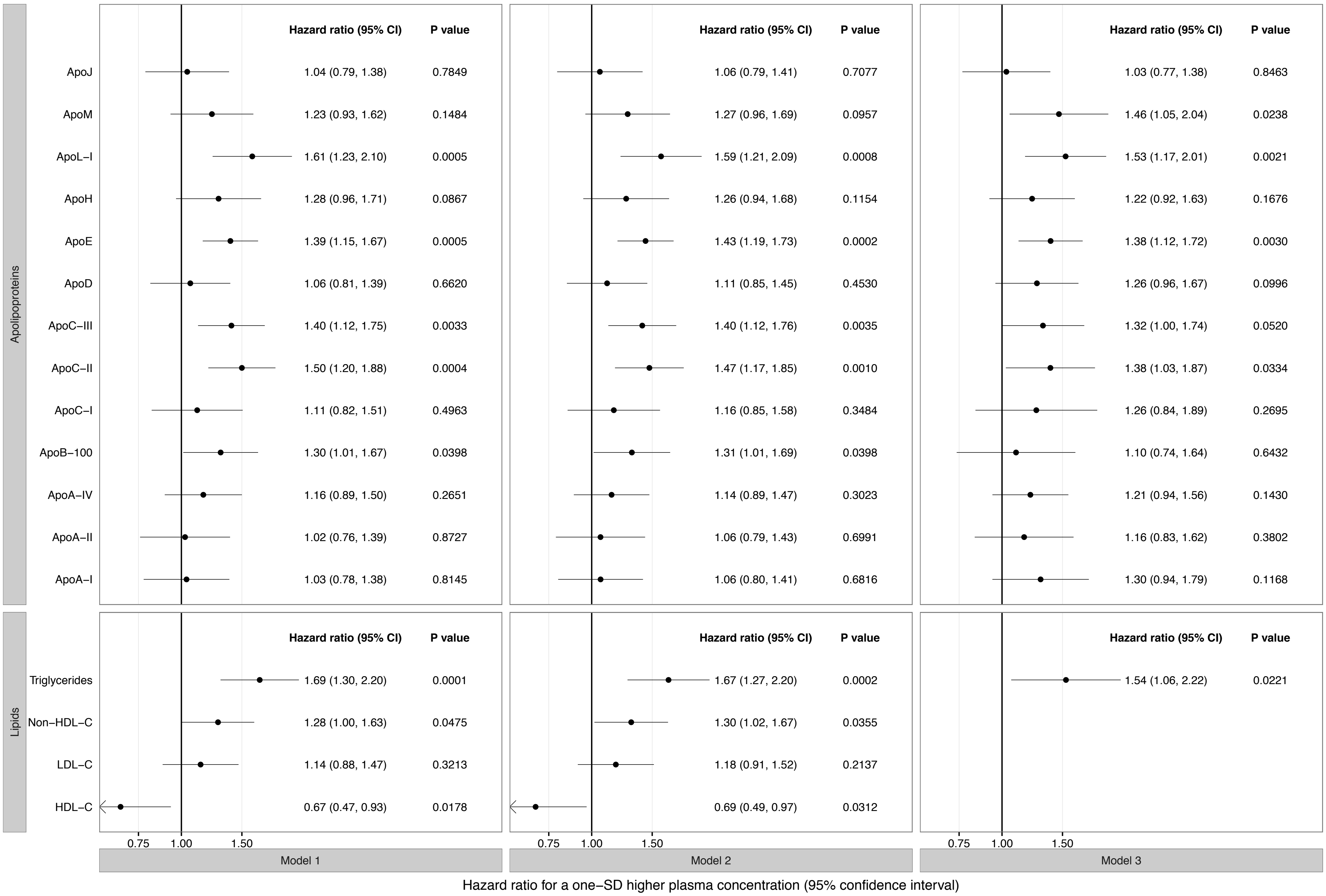

Online Figure 1B

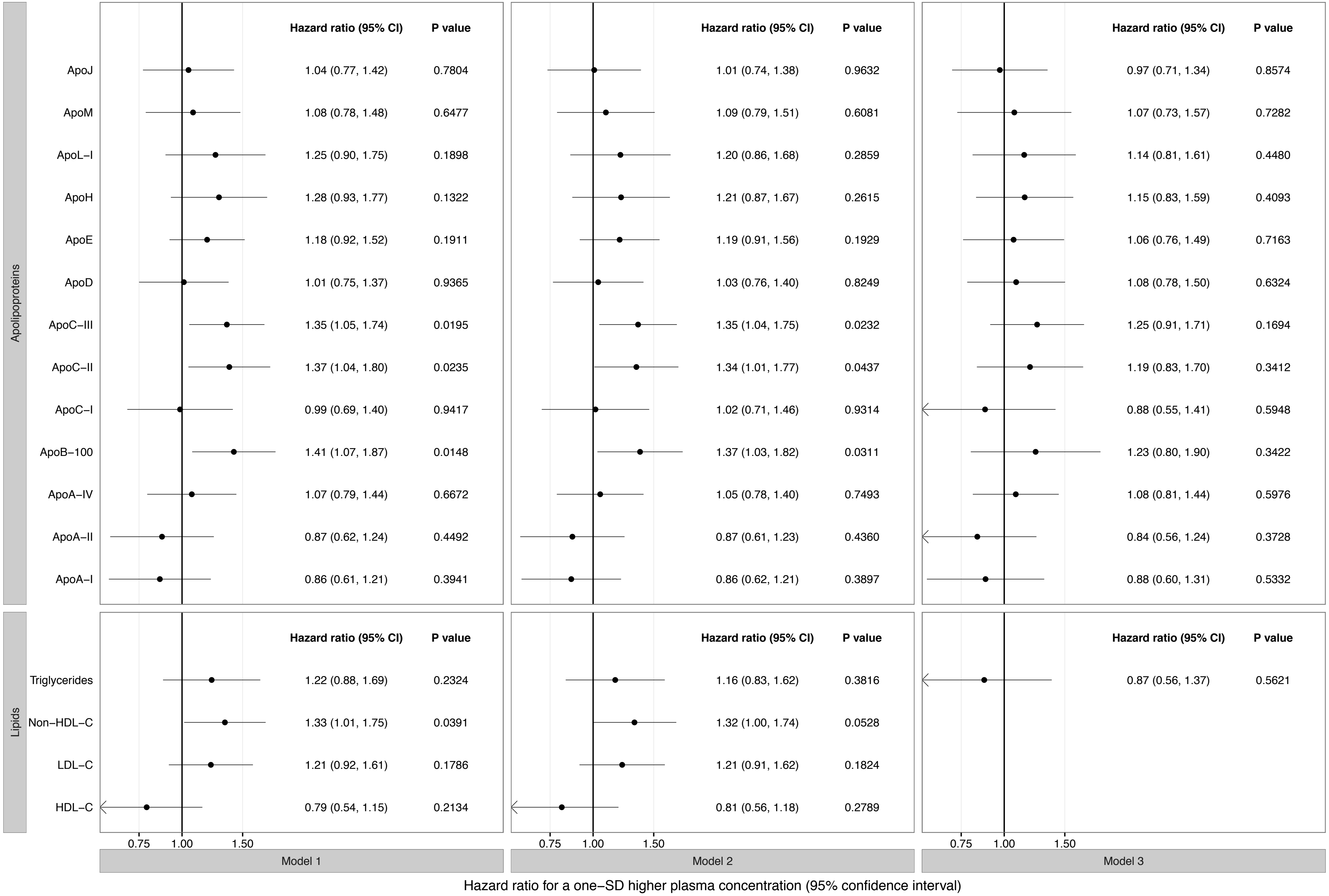

Online Figure 2

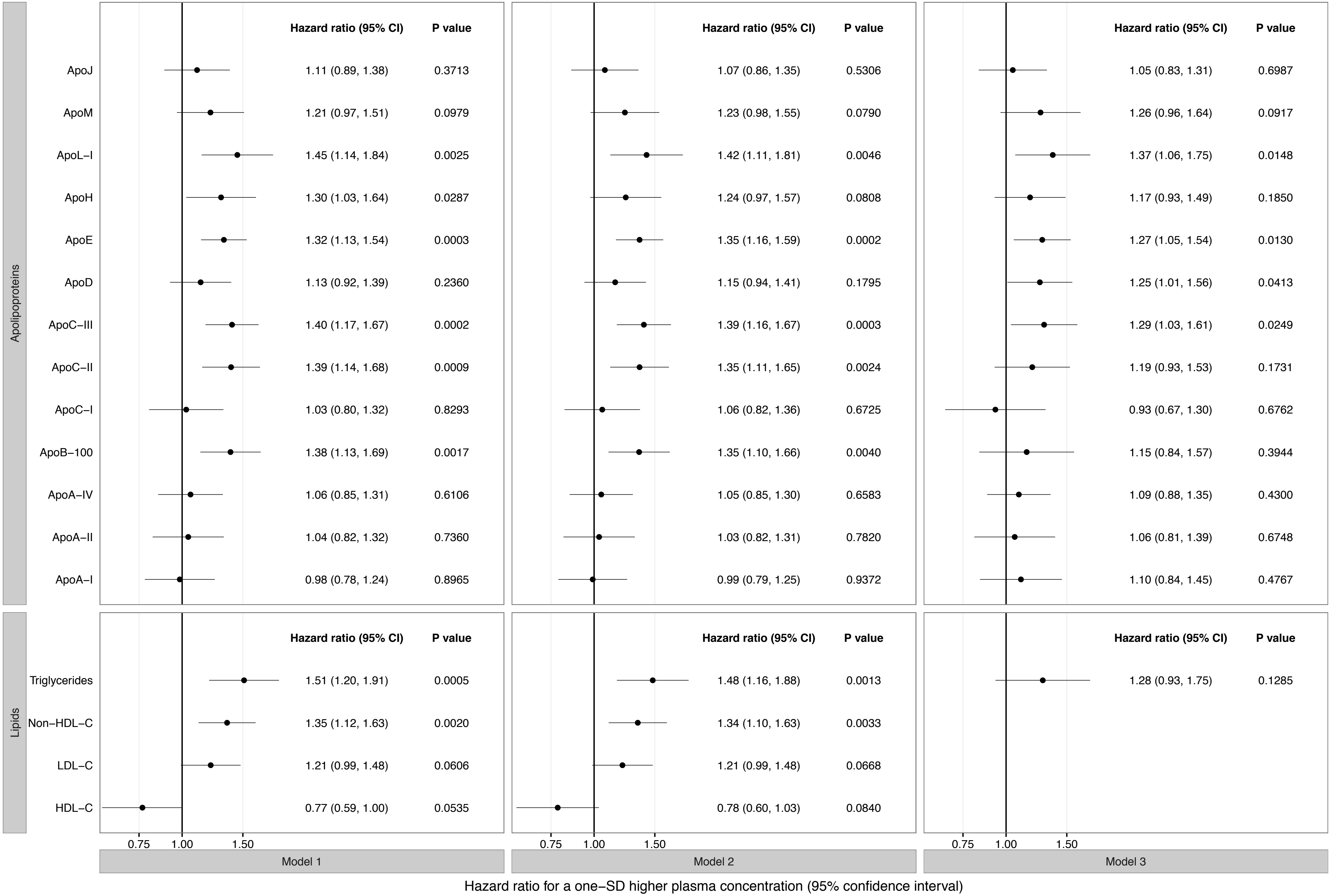

Online Figure 3

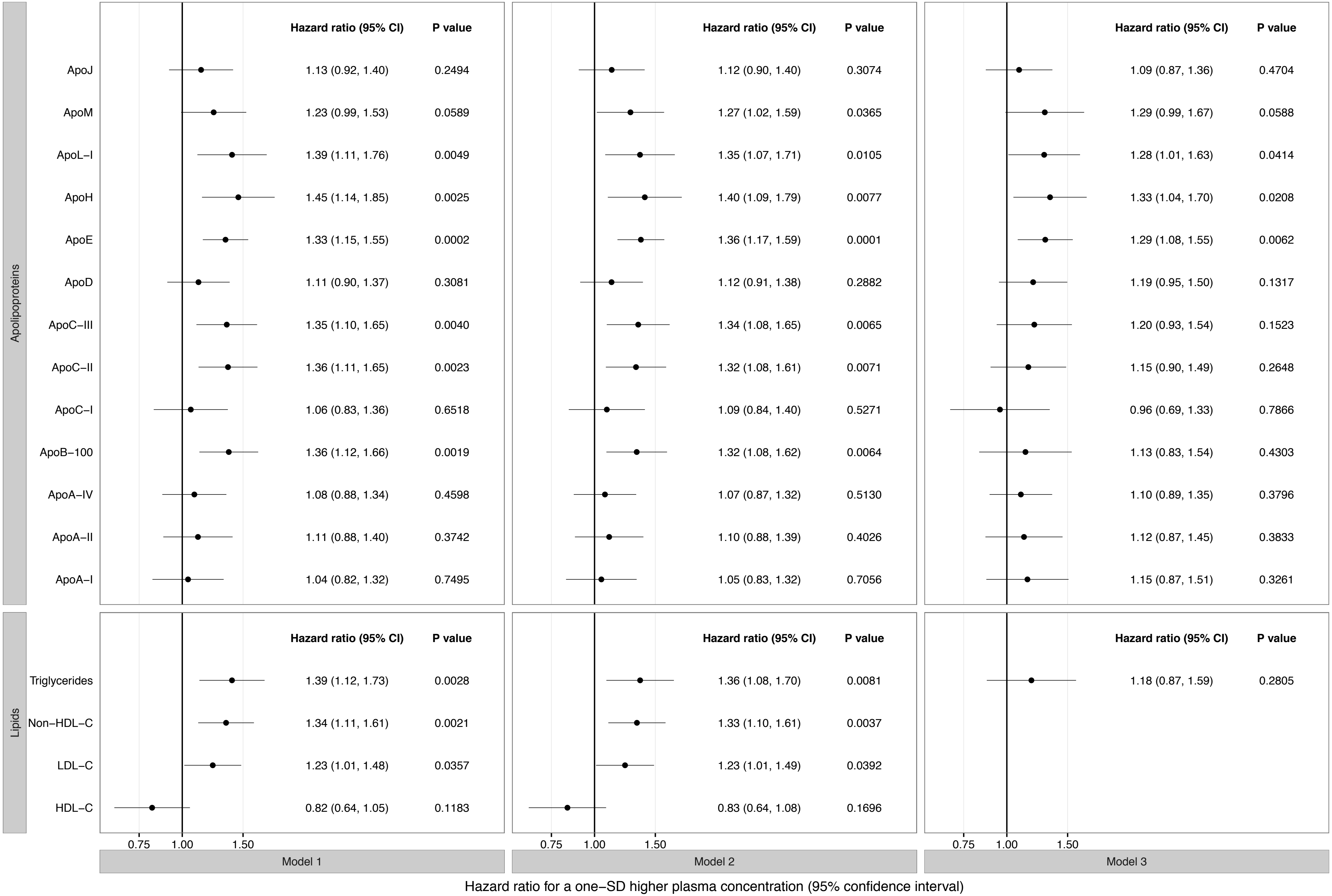

Online Figure 4

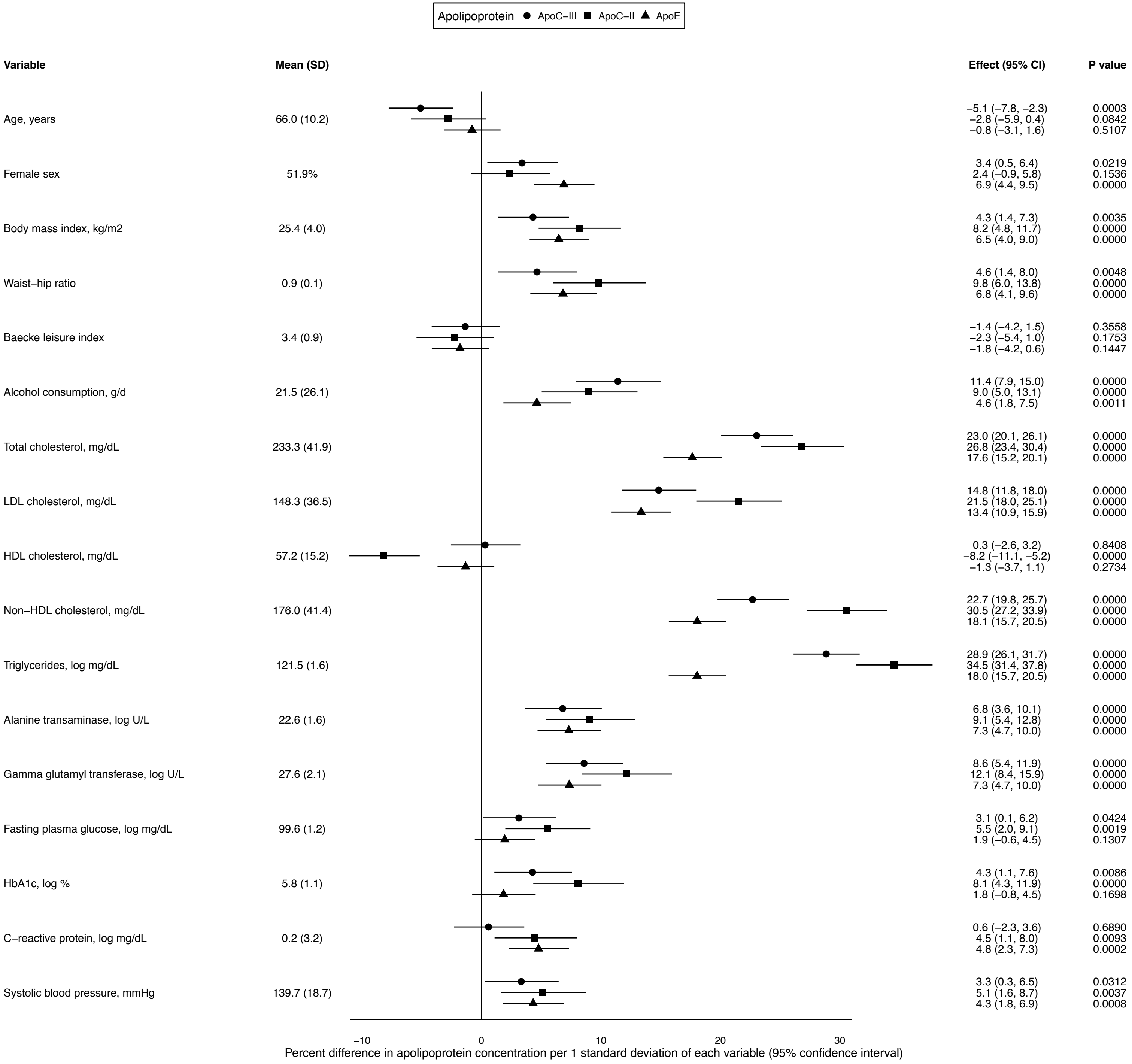

Online Figure 5A

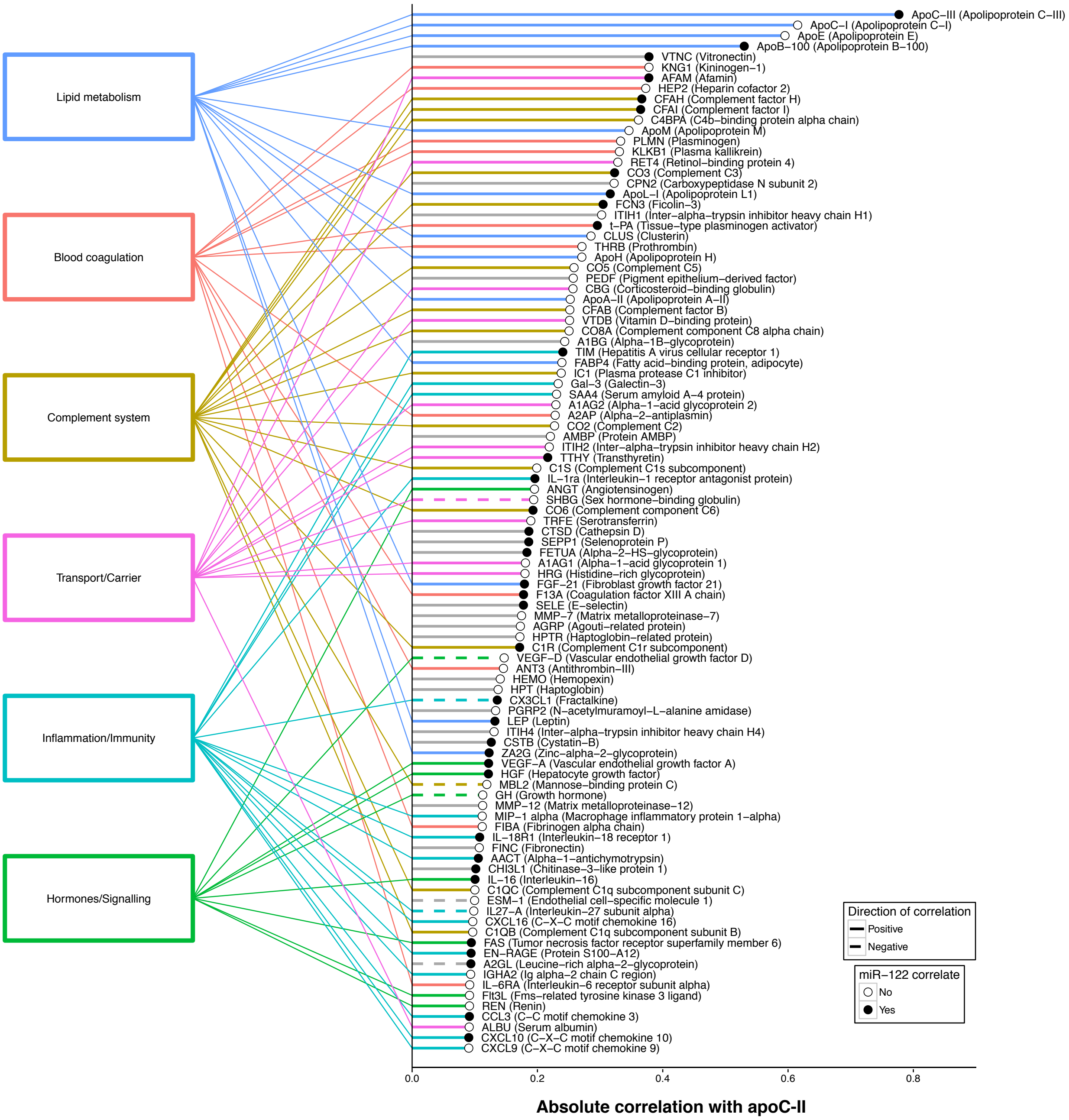

Online Figure 5B

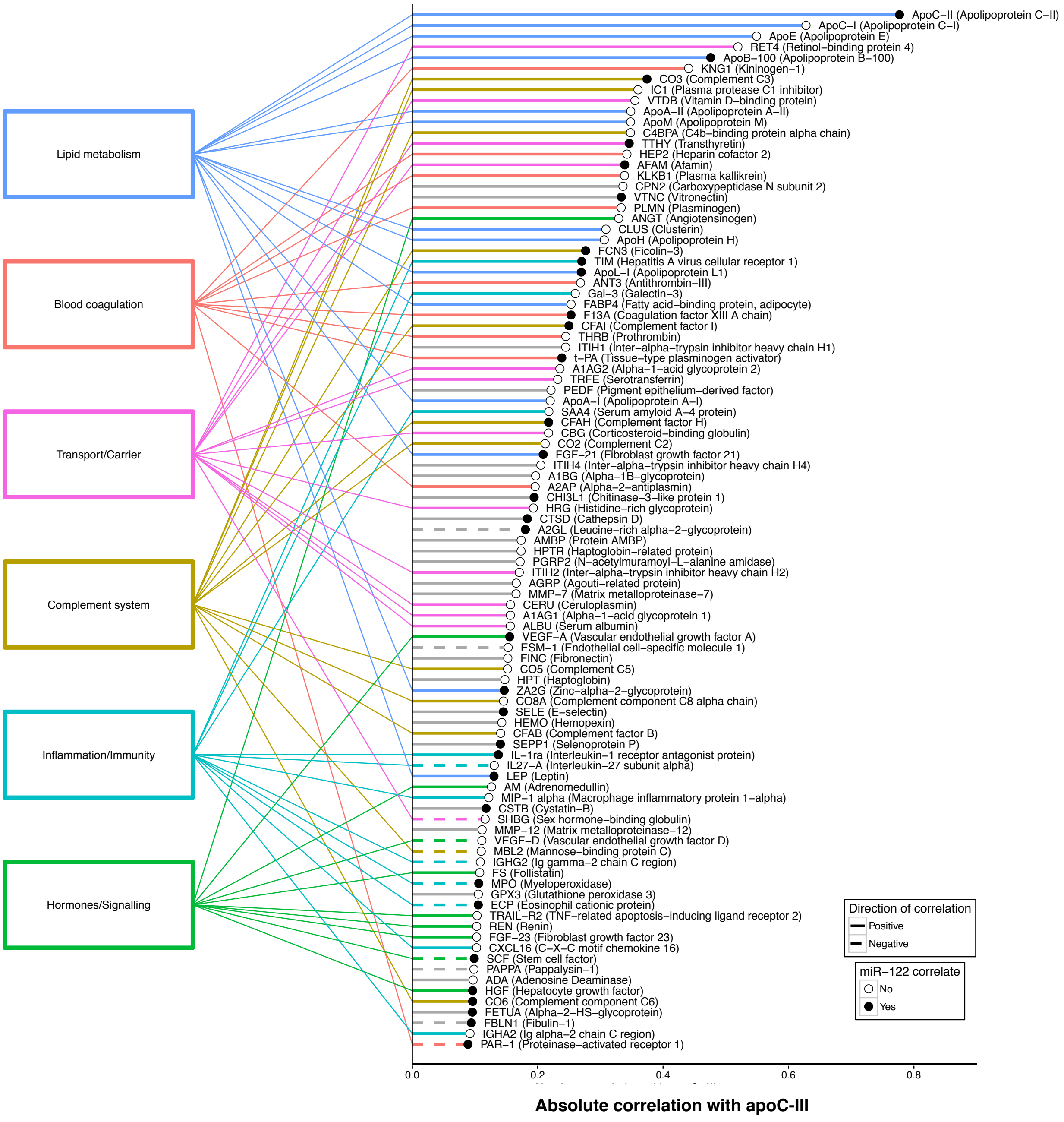

Online Figure 5C

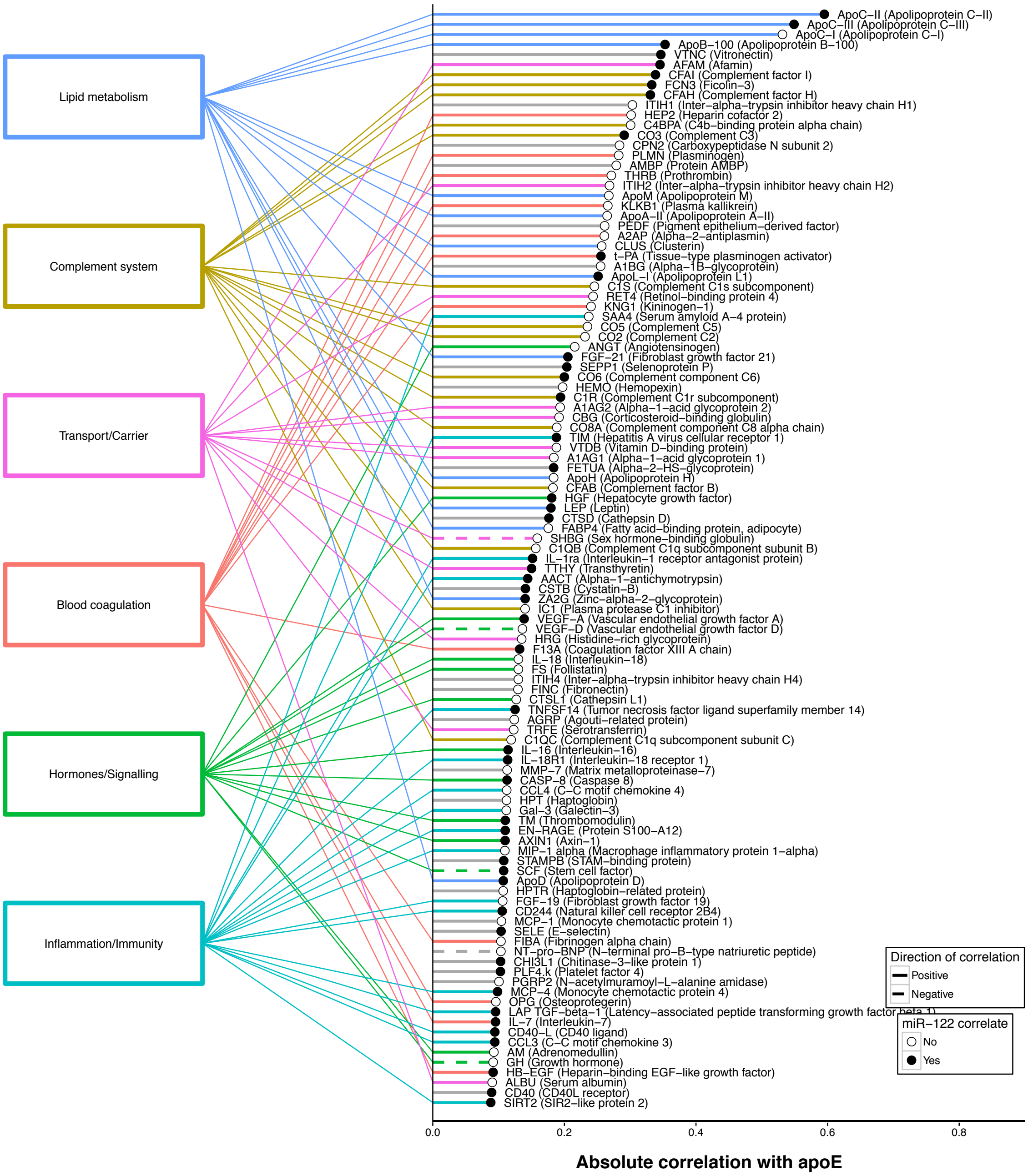

Online Figure 6A

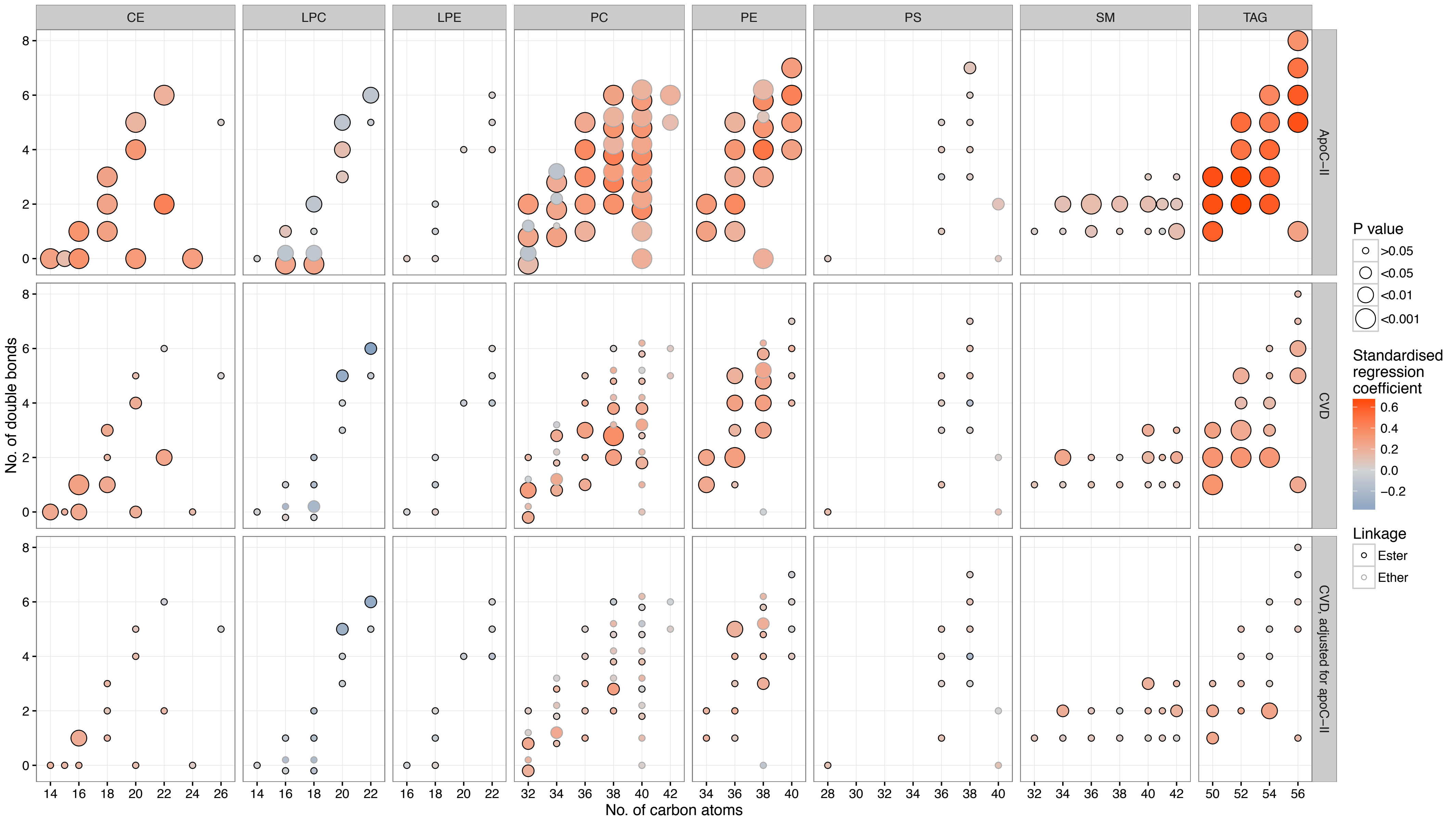

Online Figure 6B

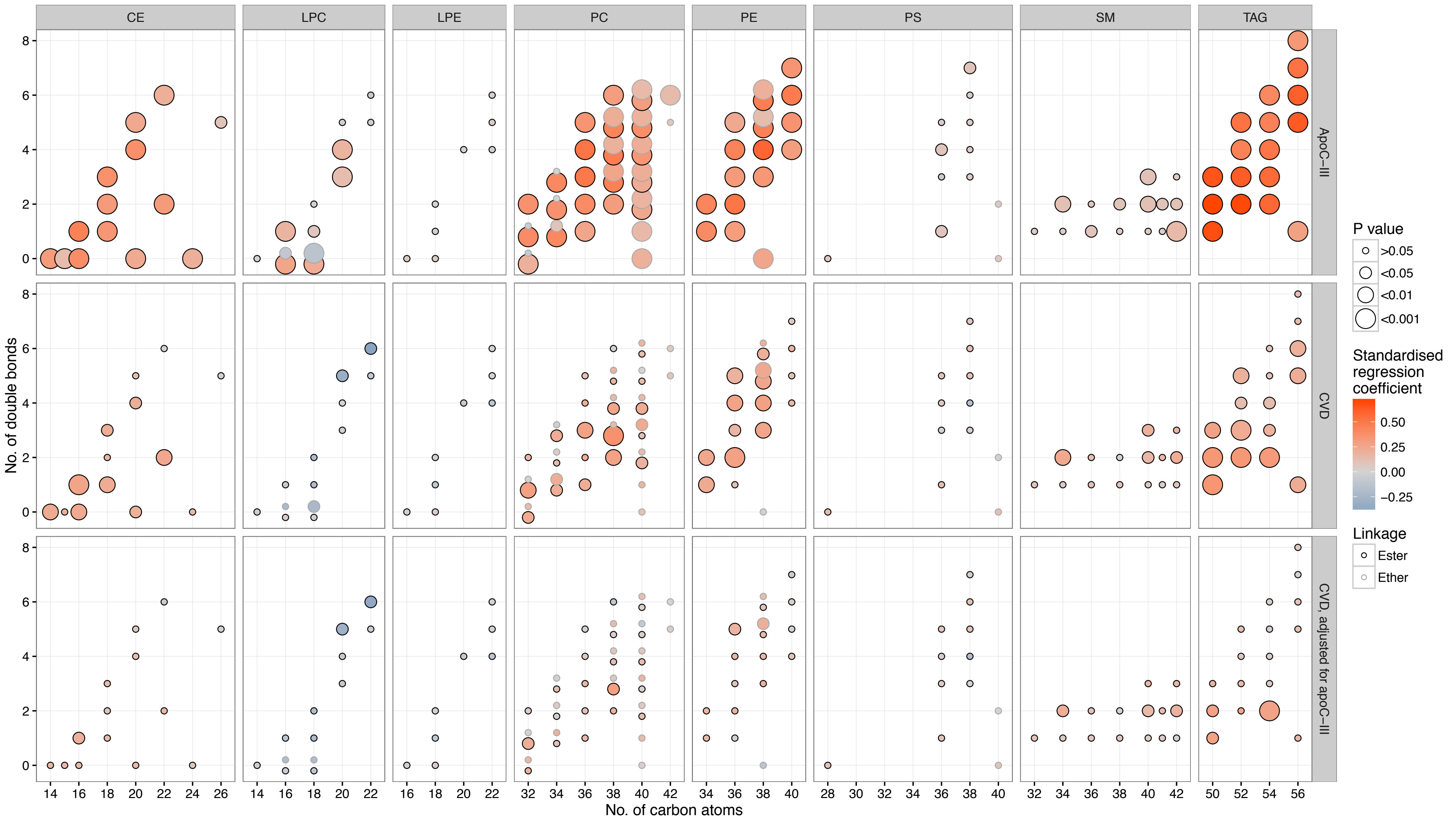

Online Figure 6C

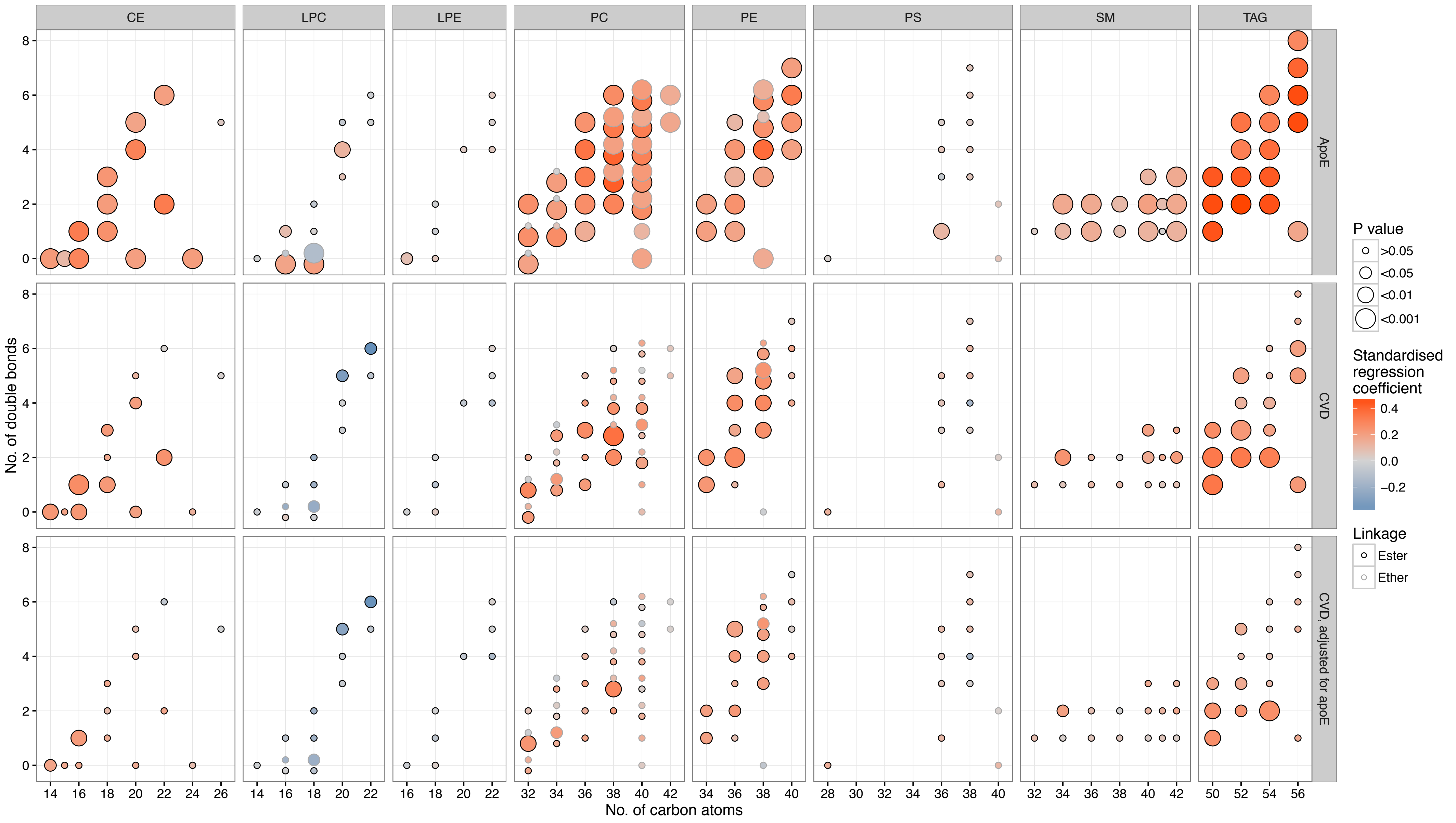

Supplement: Online Data [file mmc1.pdf]
